# Prospective epigenome and transcriptome analyses of cord and peripheral blood from preterm infants at risk of bronchopulmonary dysplasia

**DOI:** 10.1038/s41598-023-39313-0

**Published:** 2023-07-28

**Authors:** Hye-Youn Cho, Xuting Wang, Michelle R. Campbell, Vijayalakshmi Panduri, Silvina Coviello, Mauricio T. Caballero, Brian D. Bennett, Steven R. Kleeberger, Fernando P. Polack, Gaston Ofman, Douglas A. Bell

**Affiliations:** 1 Immunity, Inflammation and Disease Laboratory, National Institute of Environmental Health Sciences, National Institutes of Health, Research Triangle Park, NC 27709 USA; 2 Epigenetics and Stem Cell Biology Laboratory, National Institute of Environmental Health Sciences, National Institutes of Health, Research Triangle Park, NC 27709 USA; 3 Fundación INFANT, Buenos Aires, Argentina; 4 Consejo Nacional de Investigaciones Científicas y Técnicas (CONICET), Buenos Aires, Argentina; 5 Biostatistics and Computational Biology Branch, National Institute of Environmental Health Sciences, National Institutes of Health, Research Triangle Park, NC 27709 USA; 6 Department of Pediatrics, Vanderbilt University Medical Center, Nashville, TN 37232 USA; 7 Section of Neonatal-Perinatal Medicine, Center for Pregnancy and Newborn Research, University of Oklahoma Health Sciences Center, Oklahoma City, OK 73104 USA; 8 Immunity, Inflammation and Disease Laboratory, National Institute of Environmental Health Sciences, Building 101, MD C3-03, 111 TW Alexander Dr., Research Triangle Park, NC 27709 USA

**Keywords:** Developmental biology, Genetics, Biomarkers, Diseases, Molecular medicine, Pathogenesis

## Abstract

Bronchopulmonary dysplasia (BPD) is a prevalent chronic lung disease of prematurity with limited treatment options. To uncover biomarkers of BPD risk, this study investigated epigenetic and transcriptomic signatures of prematurity at birth and during the neonatal period at day 14 and 28. Peripheral blood DNAs from preterm infants were applied to methylation arrays and cell-type composition was estimated by deconvolution. Covariate-adjusted robust linear regression elucidated BPD- and prolonged oxygen (≥ 14 days) exposure-associated CpGs. RNAs from cord and peripheral blood were sequenced, and differentially expressed genes (DEGs) for BPD or oxygen exposure were determined. Estimated neutrophil–lymphocyte ratios in peripheral blood at day 14 in BPD infants were significantly higher than nonBPD infants, suggesting an heightened inflammatory response in developing BPD. BPD-DEGs in cord blood indicated lymphopoiesis inhibition, altered Th1/Th2 responses, DNA damage, and organ degeneration. On day 14, BPD-associated CpGs were highly enriched in neutrophil activation, infection, and CD4 + T cell quantity, and BPD-DEGs were involved in DNA damage, cellular senescence, T cell homeostasis, and hyper-cytokinesis. On day 28, BPD-associated CpGs along with BPD-DEGs were enriched for phagocytosis, neurological disorder, and nucleotide metabolism. Oxygen supplementation markedly downregulated mitochondrial biogenesis genes and altered CpGs annotated to developmental genes. Prematurity-altered DNA methylation could cause abnormal lymphopoiesis, cellular assembly and cell cycle progression to increase BPD risk. Similar pathways between epigenome and transcriptome networks suggest coordination of the two in dysregulating leukopoiesis, adaptive immunity, and innate immunity. The results provide molecular insights into biomarkers for early detection and prevention of BPD.

## Introduction

Bronchopulmonary dysplasia (BPD) is a prominent chronic respiratory disease among survivors of extremely preterm (< 28 weeks of gestational age, GA) and very low birth weight (BW, < 1500 g) infants^1,2^. The modern consensus on BPD pathogenesis is largely attributed to the arrested lung development due to premature birth which interrupts the intra-uterine programmed lung development and the subsequent repair of lung injury caused by the supplemental oxygen (O_2_) and mechanical ventilation in the neonatal intensive care unit (NICU)^2^. Lack of definitive cure and the persistent lung impairment can lead to long-term pulmonary dysfunction and increased risk for adverse respiratory symptoms (e.g., airway diseases, exercise intolerance) in BPD survivors^3–6^.

There are limited genetic, epigenetic, or transcriptomic resources for the investigation of BPD etiology and pathogenesis in preterm infants. In previous studies, genome-wide association studies (GWASs) and exome sequencing in various populations with BPD elucidated potential genetic risk factors such as *SPOCK2* encoding SPARC (osteonectin), Cwcv and Kazal like domains proteoglycan 2 and CRP encoding C-reactive protein^7–12^. Transcriptomic studies by database search, microarray, and RNA sequencing (RNA-Seq) have reported the differentially expressed genes (DEGs) in blood, lung cell or lung biopsy from BPD cases^13–17^. Most recently single cell RNA-Seq determined monocyte (tracheal aspirate)- or CD8 + T cell-specific BPD-associated genes^18,19^. Epigenome-wide association studies (EWASs) have identified DNA methylation loci associated with BPD in formalin-fixed lung autopsy/biopsy^15^ or with neonatal morbidities including BPD^20^. Recently, we reported epigenetic markers of BPD risk in cord blood DNAs from a preterm cohort^21^, and they included CpGs annotated with genes including cathepsin H (*CTSH*, cg24847366) and *SPOCK2* (cg17958658)^22^. The results indicated that cord blood methylome changes involved in lung and tissue development, cell cycle and leukopoiesis, immune-mediated inflammation, T and B cell responses, and platelet activation may precede or indicate risk of BPD development^22^. In that report, we also demonstrated that methylation-based cord blood cell-type composition varied by BW or GA, and that a higher nucleated red blood cell (NRBC) proportion was significantly associated with a lower BW and DNA hypomethylation profile^22^.

In the current study, we expanded cell-type deconvolution of DNA methylation to postnatal peripheral blood from the same preterm cohort^21,22^. Methylome analysis during the newborn period determined potential postnatal epigenome markers of BPD development and RNA-Seq demonstrated BPD-associated DEGs in cord and postnatal peripheral blood cells from the premature infants. In addition, influence of prolonged NICU O_2_ supplementation on blood cell DNA methylation and gene expression landscape was elucidated in nonBPD infants on postnatal day 14. This investigation of the early life hematologic epigenome and transcriptome provides further insights and biomarkers of BPD pathogenesis.

## Results

### Demographics of cohort

A total of 109 premature infants were included in the study after applying exclusion criteria. Table 1 and Additional file 1: Table S1 present the neonatal and maternal characteristics and fetal complications of the prematurely born neonates diagnosed with BPD or without (nonBPD). Diagnosis of BPD was made for 15 preterm infants based on the National Institutes of Health consensus definition and O_2_ needs at either 36 weeks postmenstrual age or at 28–56 days of postnatal life based on GA^23^. BPD development was sexually dimorphic with 25% of males (10/40) and 7.2% of females (5/69) diagnosed among these premature infants. Infants diagnosed with BPD were born at significantly lower mean BW (*p* = 0.002) and GA (*p* < 0.001) and were supplemented with NICU O_2_ for a greater number of days (46.5 ± 4.0, *p* < 0.001) compared to nonBPD infants (7.8 ± 1.1). Infections were more common in the neonates who developed BPD (Table 1, *p* = 0.006). The time points (14 days and 28 days of life) used for peripheral blood analysis were chosen preceding symptoms and diagnosis of BPD.

**Table 1:** Characteristics of the preterm infant cohort used for overall analyses.

Characteristics	BPD	nonBPD	*p* value
Neonate characteristics			
Sample size	15	94	
Birth weight (g)	938.8 ± 71.5	1187.6 ± 25.0	0.002
Gestational age (weeks)	27.5 ± 0.6	30.2 ± 0.2	< 0.001
Sex	Male 10 (67%)	Male 30 (32%)	0.013
	Female 5 (33%)	Female 64 (68%)	
Cumulative NICU O_2_ (Days)	46.5 ± 4.0	7.8 ± 1.1	< 0.001
Day	0	31	
1–13 days	0	40	
≥ 14 days	15	22	
Unknown	0	1	
Delivery room surfactant	1/13 (8%)	4/87 (5%)	0.644
Infection in NICU	8/14 (57%)	20/93 (22%)	0.006
Maternal characteristics			
Maternal ancestry			0.863
European-Latin	12	69	
Criollos	0	14	
European-other	0	5	
Jewish	1	3	
African Caribbean	0	1	
Asian	0	1	
Native American	1	0	
Arab-Middle Eastern	1	0	
Unknown or refused	0	1	
Maternal age (year)	32.2 ± 1.9	34.1 ± 0.7	0.369
Maternal smoking history^a^	7/15 (47%)	25/94 (27%)	0.116
Maternal antenatal steroid	15/15 (100%)	83/91 (91%)	0.239
Maternal pre-eclampsia	3/15 (20%)	26/94 (28%)	0.539
Gestational diabetes	0/14 (0%)	1/90 (1%)	0.714
BMI	34.6 ± 3.1	35.6 ± 1.3	1.000
Education^b^	10/4/0/1	63/20/5/6	0.916
Alcohol during pregnancy	0/13 (0%)	1/89 (1%)	0.724
Chorioamnionitis	2/15 (13%)	5/94 (5%)	0.246
Fetal complications			
Fetal IUGR	5/13 (39%)	38/89 (43%)	0.778
Fetal oligohydramnios	3/12 (25%)	11/89 (12%)	0.240

Samples recruited from the Discovery-Bronchopulmonary Dysplasia Program (D-BPD) cohort in Buenos Aires, Argentina^21^. Mean ± S.E.M. presented. *NICU* neonatal intensive care unit, *O*_*2*_ oxygen supplement, *IUGR* intrauterine growth restriction. Detailed cohort characteristics are in Additional file: Table S1. ^a^Self-reported former and/or current smoker. ^b^Higher/Secondary/Primary/NA.

### Methylation-based estimation of blood cell composition

**Figure 1:**
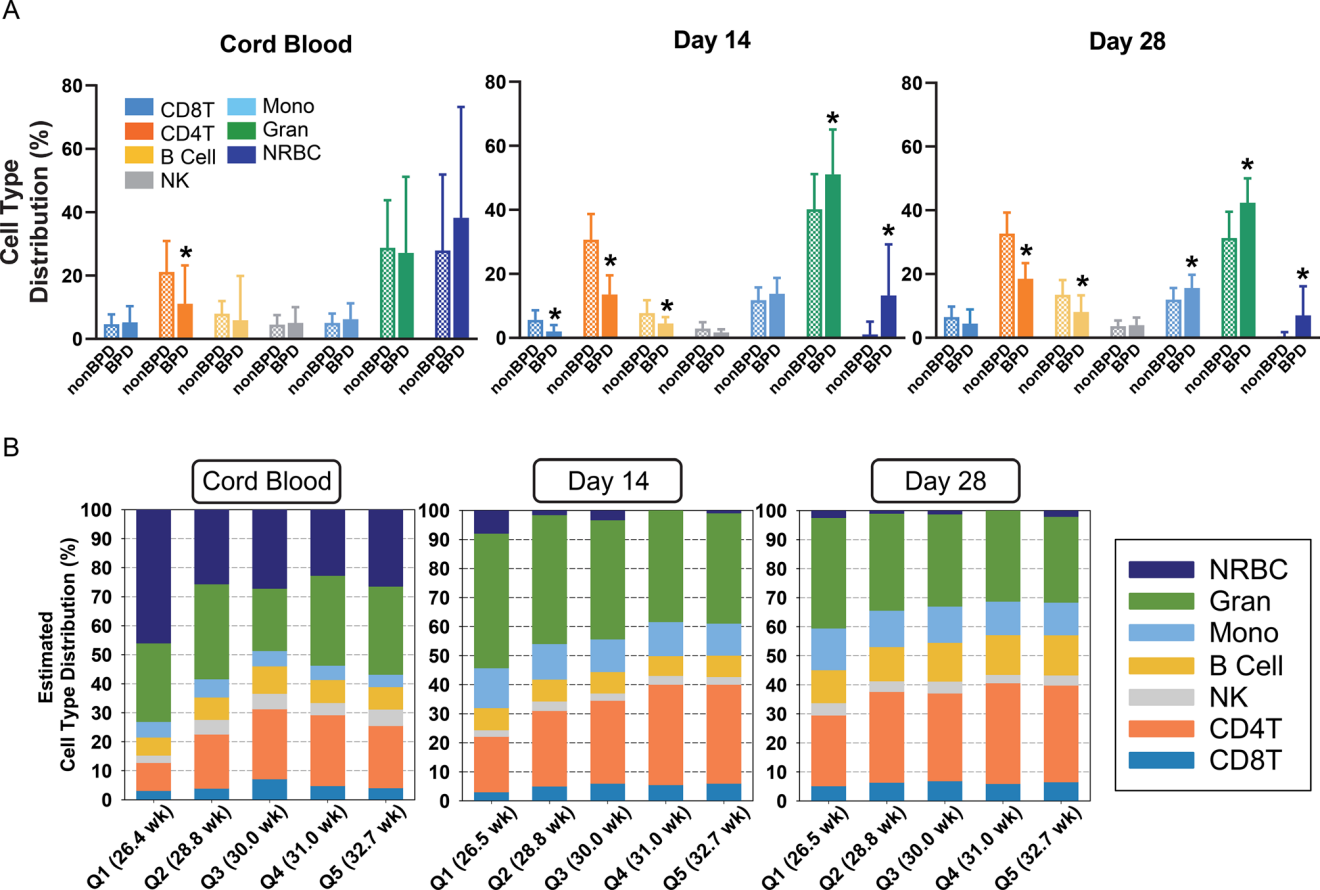
DNA methylation-based estimation of blood cell type composition in preterm infants. (**A**) Seven cell-type distributions in preterm infants with bronchopulmonary dysplasia (BPD) development or without (nonBPD) were estimated by DNA methylation profiles of cord blood and peripheral blood on postnatal days 14 and 28. Individual cell type data is in Additional file: Table S1. Mean ± S.E.M. presented. *p < 0.05 vs. time-matched nonBPD. (**B**) Blood cell type distribution of all preterm infants by gestational age quintile (Q). *CD4T* CD4 + T cells, *CD8T* CD8 + T cells, *NK* natural killer cells, *Mono* monocytes, *Gran* granulocytes, *NRBC* nucleated red blood cell. n = 107 in cord blood (93 nonBPD, 14 BPD). n = 93 on day 14 (81 nonBPD, 12 BPD). n = 82 on day 28 (71 nonBPD, 11 BPD).

The methylation-based deconvolution model estimated seven cell type percentages in cord and peripheral blood. Of these, six cell types (with the exception of natural killer (NK) cells) were determined to be significantly varied between BPD and nonBPD neonates at one or more time points (Fig. 1A). The percentage of lymphocytes including CD8 + T cells (day 14), CD4 + T cells (cord blood, days 14 and 28), and B cells (days 14 and 28) were significantly decreased in BPD relative to nonBPD (Fig. 1A). In contrast, percentage of myeloid cells including monocytes (day 28) and granulocytes (days 14 and 28) and NRBCs (days 14 and 28) were significantly higher in BPD than in nonBPD (Fig. 1A). The previously reported cord blood cell-type compositions^22^ based on the Houseman deconvolution algorithm^24^ were re-evaluated together with postnatal day data using the approach of Koestler et al. (Identifying Optimal DNA methylation Libraries, IDOL)^25^.

The estimated seven cell-type distribution plotted relative to GA quintiles among all premature infants indicated time- and GA-dependent shifts of cell-type composition during the neonatal period (Fig. 1B). Compared to cord blood, NRBC proportions in peripheral blood from days 14 and 28 were markedly decreased in all GA quintile groups (Fig. 1B). Higher CD4 + T cell percentage in higher GA groups were also evident at all times and a similar trend was shown for percentage of CD8 + T cells on day 14 and percentage of B cells in cord blood and on day 28 (Fig. 1B). In contrast granulocyte proportions, assumed to be largely neutrophils, were higher in lower GA groups on days 14 and 28 (Fig. 1B).

**Figure 2:**
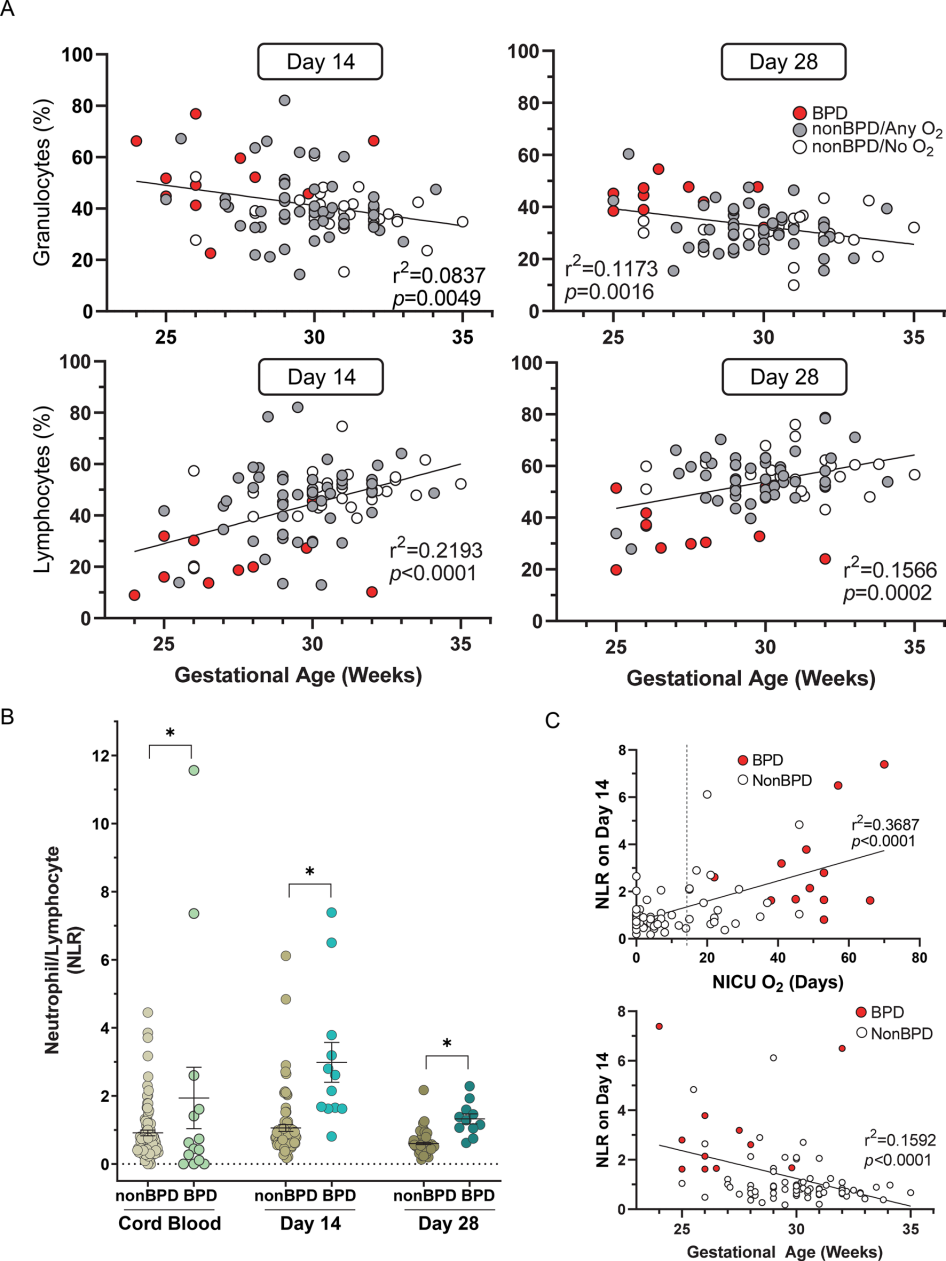
Association between granulocytes, lymphocytes, and gestational age (GA) in preterm neonates diagnosed with bronchopulmonary dysplasia (BPD) or without (nonBPD). (**A**) Significant correlations between GA and peripheral blood granulocyte, or total lymphocyte, depicted by disease status and oxygen (O_2_) exposure days in neonatal intensive care unit (NICU). (**B**) Significantly elevated neutrophil (granulocyte)-lymphocyte ratio (NLR) in BPD compared to nonBPD in cord blood and peripheral blood on postnatal days 14 and 28. *, *p* < 0.05 vs. time-matched nonBPD. Individual data with mean ± S.E.M. presented. n = 107 in cord blood (93 nonBPD, 14 BPD). n = 93 in day 14 (81 nonBPD, 12 BPD). n = 82 in day 28 (71 nonBPD, 11 BPD). (**C**) Higher NLR was also associated with prolonged NICU O_2_ days and shorter GA on day 14 and  postnatal day 28 (Additional file 2: Fig. S1 A, B).

There were significant inverse correlations between GA and granulocyte percentage on both postnatal days in preterm infants (day 14, r^2^ = 0.0837, *p* = 0.0049; day 28, r^2^ = 0.1173, *p* = 0.0016) (Fig. 2A). GA was also significantly, but positively, associated with the percentage of the sum of all lymphoid subpopulations (CD4 + T, CD8 + T, B cell, NK) on both postnatal days (day 14 r^2^ = 0.2193, *p* = 1.0E−04; day 28, r^2^ = 0.1566, *p* = 2.0E−04) (Fig. 2A). An increased percentage of granulocytes and a suppressed percentage of lymphocytes was observed in BPD infants relative to nonBPD infants (Fig. 2A). The neutrophil (granulocyte)-lymphocyte ratio (NLR), reported to be an early predictor of BPD^26,27^, was significantly higher in BPD compared to nonBPD on all neonatal days (Fig. 2B). Examining NLR, we found significant correlations with NICU O_2_ days and GA (Fig. 2C). NLRs were lowered by day 28 (Fig. 2B) while the significant positive correlation to NICU O_2_ days persisted (Additional file 2: Fig. S1). Overall we observed that higher NLR was correlated with lower GA, longer O_2_ supplementation, and associated with development of BPD.

To further explore differences in cell-type proportions, particularly shifts among lymphocyte subtypes from naïve to memory, we utilized the 12 cell-type model of Salas et al.^28^. While this model does not include NRBCs and cannot distinguish NRBCs from myeloid lineage cell types, it estimates naïve and memory composition of B cells, CD4 T cells and CD8 T cells. BPD infants showed significant increases in memory B cells at both day 14 and day 28 (*p* < 0.01, Additional file 2: Table S2), significant decreases in CD4 and CD8 naïve cell types at day 14 (*p* < 0.05). This model also indicated a highly significant increase in NLR for BPD infants at both day 14 and day 28 (*p* < 0.01).

### Cord blood genes differentially expressed in BPD and comparison with EWAS

**Figure 3:**
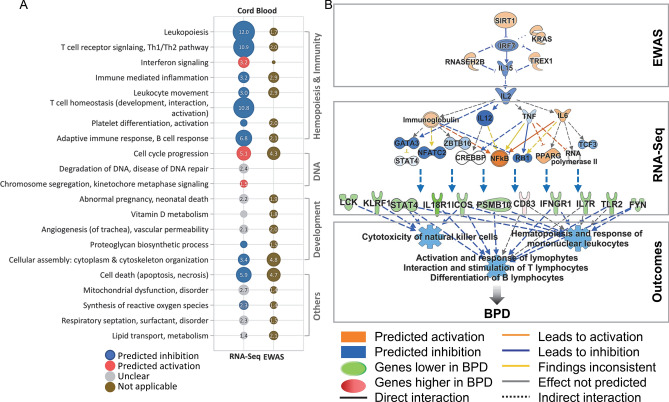
Bronchopulmonary dysplasia (BPD)-associated cord blood genes, predicted pathways and comparison with cord blood epigenetic changes. (**A**) A dot plot of highly enriched pathways and gene ontologies (GOs) of differentially expressed cord blood genes (471 genes at *p* < 0.01) in BPD (n = 12) compared to nonBPD (n = 55, ≥ 1 day oxygen-exposure in newborn intensive care unit) determined by RNA sequencing analysis (RNA-Seq) with adjustment for covariates (6 cell-type %, sex, gestational age, birth weight). Top-ranked pathways of cord blood epigenome-wide association analysis (EWAS) for BPD (386 genes annotated to 275 CpGs) were retrieved from a previous publication^22^. Circle size and number represent adjusted -Log_10_ *p* values of pathways/GOs. Circle color indicates activation z-score trend. Details of pathway analysis are in Additional file: Table S4. (**B**) Ingenuity pathway analysis (IPA) demonstrates that interleukin 2 (IL-2) may be an essential upstream molecule disturbing epigenomes and transcriptomes involved in blood cell development and activity during BPD pathogenesis. *Sirt1* sirtuin 1, *TREX1* three prime repair exonuclease 1, *IRF2* interferon regulatory factor 2, *RNASEH2B* ribonuclease H2 subunit B, *TNF* tumor necrosis factor, *ZBTB16* zinc finger and BTB domain containing 16, *TCF3* transcription factor 3, *NFATC2* nuclear factor of activated T cells 2, *STAT4* signal transducer and activator of transcription 4, *CREBBP* CREB binding protein, *NFκB* nuclear factor kappa-light-chain-enhancer of activated B cells, *PPARG* peroxisome proliferator activated receptor gamma, *KLRF* killer cell lectin like receptor F1, *IL18R1* IL-18 receptor 1, *ICOS* inducible T cell costimulatory, *PSMB10* Proteasome 20S Subunit Beta 10, *IFNGR1* interferon gamma receptor 1, *TLR2* toll-like receptor 2.

RNA-seq was carried out on cord blood RNA, and DESeq2 analysis adjustment for nine covariates (GA, BW, sex, and six cell-type percentages of CD4 + T, CD8 + T, B cell, granulocyte, monocyte, and NRBC) determined DEGs in BPD infants (471 genes at *p* < 0.01; 1685 genes at *p* < 0.05) (Table 2; Additional file 1: Table S3; Additional file 2: Fig. S2A). Pathway analysis indicated that 471 DEGs were predominantly involved in the inhibition of lymphopoiesis, T cell receptor signaling and Th1/Th2 pathways, and adaptive immune response (e.g., *CD27*, *CD79B*, *ICOS*, *IL18R1*, *LCK*, *STAT4*) (Fig. 3A; Table 2; Additional file 1: Table S4), consistent with the decreased T and B cell quantity in BPD infants as depicted in Fig. 1A. The observation of the decreased pyroptosis pathway (e.g., *CASP9*, *GZMA*), activated antiviral response and interferon (IFN) signaling (e.g., *IFIM3*, *SOCS1*, *TLR4*) and mitotic cell cycle (e.g., *BORA*, *RAD51*, *SPC25*) (Fig. 3A; Additional file 1: Table S4) coordinately indicated inflammatory cell proliferation against infection, agreeing with the increased NLR found in BPD infants (shown in Fig. 2B). These and other pathways including development and respiratory disorders showed similar enrichment in the cord blood BPD-EWAS CpGs^22^. Parallel analysis of epigenome and transcriptome pathways in cord blood cells suggests an epigenetic modulation of the T cell autocrine lymphokine IL-2^29^, which could be an upstream event to manage transcriptional changes for T cell clonal expansion and growth, leukocyte activation, and IL-18 stimulation for IFN production and NK cell cytotoxicity (Fig. 3B). The 134 genes in common between BPD-CpGs and BPD-DEGs (e.g., *CCL5*, *HLA-DQA2*, *IL18RAP*, *LCK*, *SELP*) may play key roles in granulocyte inflammation and innate and adaptive immune responses (Additional file 1: Table S3; Additional file 2: Fig. S2B). Lung alveolarization- and BPD-associated *SPOCK2*^7,30^ and *AGER* encoding receptor for advanced glycosylation end-product (RAGE)^31,32^ were also elucidated in both analyses. Many of the overlapping genes showed reciprocal changes in methylation and gene expression differences indicating epigenetic regulation of their transcription.

**Table 2:** Differentially expressed cord blood genes in bronchopulmonary dysplasia (BPD).

Functions and Pathways	Gene	*p*	FC	Description
Immunity	*BCL2*	2.80E−03	− 1.90	BCL2 apoptosis regulator
	*CCR7*	1.68E−02	− 1.83	C–C motif chemokine receptor 7
	*CD247*	3.43E−02	− 1.54	CD247 molecule
	*CD27*	5.80E−03	− 1.93	CD27 molecule
	*CD28*	2.73E−02	− 1.63	CD28 molecule
	*CD79B*	1.80E−02	− 1.95	CD79b molecule
	*HLA-DQA2*†	1.82E−02	− 3.03	Major histocompatibility complex, class II, DQ alpha 2
	*ICOS*	9.42E−03	− 1.81	Inducible T cell costimulator
	*IGLV2-14*	1.24E−02	− 2.25	Immunoglobulin lambda variable 2–14
	IL18R1*	3.61E−05	− 3.35	Interleukin 18 receptor 1
	*IL18RAP*^†^	4.08E−03	− 1.97	Interleukin 18 receptor accessory protein
	*IL21R*	1.80E−02	− 2.34	Interleukin 1 receptor type 2
	*LCK*^†^	7.30E−03	− 1.83	LCK proto-oncogene, Src family tyrosine kinase
	*PGLYRP2*	2.31E−02	8.32	Peptidoglycan recognition protein 2
	*RAG2*	1.56E−02	8.96	Recombination activating 2
	*STAT4*	5.13E−03	− 1.88	Signal transducer and activator of transcription 4
	*TRBJ1-1*	1.52E−02	− 4.72	T cell receptor beta joining 1–1
	*TRAV16*	7.99E−03	− 3.93	T cell receptor alpha variable 16
	*ZAP70*	1.05E−02	− 1.93	Zeta chain of T cell receptor associated protein kinase 70
Immune-mediated inflammation, leukocyte migration, interferon signaling	*CD83*	7.56E−03	1.88	CD83 molecule
	*CXCL8*	4.02E−02	3.96	C–X–C motif chemokine ligand 8
	*GBP3*	6.74E−03	− 1.93	Guanylate binding protein 3
	*IFITM3*^†^	1.91E−02	− 1.90	Interferon induced transmembrane protein 3
	*ITGA6*	3.32E−02	− 1.64	Integrin subunit alpha 6
	*MAPK9*	3.59E−02	1.43	Mitogen-activated protein kinase 9
	SELP^†^	3.22E−02	− 2.21	Selectin P
	*SOCS1*	1.23E−02	− 2.22	Suppressor of cytokine signaling 1
	*TLR4*	1.13E−02	− 1.89	Toll like receptor 4
	*TNFSF8*	6.95E−04	− 2.14	TNF superfamily member 8
Mitosis, chromosome segregation, DNA repair	*AURKB*	1.88E−02	1.85	Aurora kinase B
	BORA*	3.71E−06	1.96	BORA aurora kinase A activator
	*CDCA8*	3.11E−04	2.21	Cell division cycle associated 8
	*MKI67*	1.06E−02	1.62	Marker of proliferation Ki-67
	RAD51*	2.06E−05	3.00	RAD51 recombinase
	*SPC25*^†^	1.43E−02	2.76	SPC25 component of NDC80 kinetochore complex
	*TOP3A*	5.76E−03	1.47	DNA topoisomerase III alpha
	*WEE1*^†^	2.86E−02	1.41	WEE1 G2 checkpoint kinase
Cell death, pyroptosis	*CASP9*	5.82E−03	1.53	Caspase 9
	*GSDMD*	4.73E−02	− 1.49	Gasdermin D
	*GZMA*^†^	2.20E−03	− 1.87	Granzyme A
Respiratory disorders	*AGER*^†^	2.55E−02	1.54	Advanced glycosylation end-product specific receptor
	*CAMP*	7.17E−04	3.33	Cathelicidin antimicrobial peptide
	HBG1*	1.43E−05	− 47.85	Hemoglobin subunit gamma 1
Growth, angiogenesis	*ANGPT2*^†^	2.89E−02	5.46	Angiopoietin 2
	B4GALT5*	4.15E−06	1.92	Beta-1,4-galactosyltransferase 5
	*GPS2*	9.24E−03	− 1.88	G protein pathway suppressor 2
	MMUT*	2.00E−05	1.67	Methylmalonyl-CoA mutase
	PAPPA2*	5.09E−38	> 1000	Pappalysin 2
	*PGF*^†^	1.96E−02	5.60	Placental growth factor
	*SPOCK2*^†^	2.58E−02	− 1.91	SPARC (osteonectin), cwcv and kazal like domains proteoglycan 2
	*VEGFA*	4.71E−03	2.01	Vascular endothelial growth factor A

Differentially expressed genes (1685 genes at *p* < 0.05) between BPD (n = 12) and nonBPD (n = 55) cord blood cells determined by RNA-sequencing. Ingenuity, Reactome, and ToppGene pathway analysis tools determined by enriched ontologies and pathways. *FC* fold difference in BPD vs. nonBPD. Full list of the DEGs and pathways in Additional file: Tables S3 and S4. *Genes significant at false discovery rate 10%. ^†^Genes overlapped with those annotated to epigenome-wide association study previously published^22^.

### BPD-associated CpGs and DEGs in peripheral blood cells on postnatal day 14

**Figure 4:**
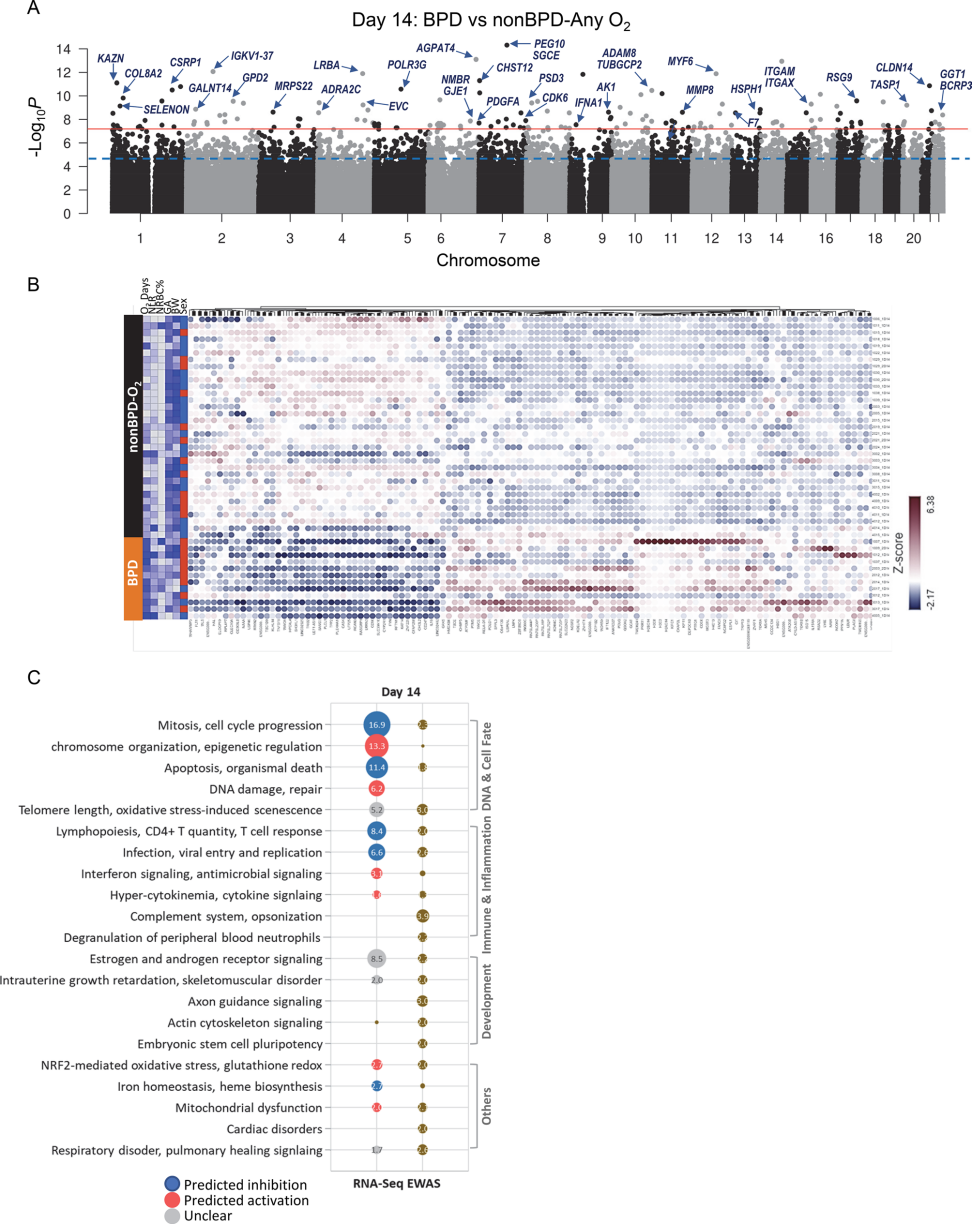
Epigenome-wide association study (EWAS) and genome-wide gene expression analysis for bronchopulmonary dysplasia (BPD) development in blood cells from premature infants on postnatal day 14. (**A**) A Manhattan plot displays BPD-associated CpGs on day 14 by comparison of BPD (n = 12) and nonBPD (n = 53) exposed to ≥ 1 day of oxygen (O_2_) in the neonatal intensive care unit (NICU) with adjustment for covariates [seven cell type %, sex, gestational age (GA), birth weight (BW)]. Robust linear regression model elucidated 153 CpGs with Bonferroni correction (*p* < 6.32E−08, red line) and 2871 CpGs at false discovery rate (FDR) 1% (blue dotted line). (**B**) Heat map depicts hierarchical clustering of differentially expressed 233 genes in BPD (FDR 10%) with adjustment for six cell type %, sex, GA, and BW determined by RNA sequencing (RNA-Seq, 12 BPD, 33 nonBPD-O_2_). Down-regulated and up-regulated genes in BPD relative to nonBPD are in blue and orange, respectively. NICU O_2_ days, neutrophil–lymphocyte ratio (NLR), nucleated red blood cell (NRBC) %, GA, and BW (color intensity = scale) as well as sex (red=male, blue=female) are labeled for each sample. Samples by row and genes by column. Heatmap created using Partek Flow (Partek Inc., Chesterfield, MO; https://www.partek.com/partek-flow/). (**C**) Top-ranked pathways and gene ontologies of BPD-associated genes and BPD CpG-annotated genes demonstrate similar or unique molecular events in transcriptome and epigenome. Circle size and label indicate –log_10_ *p* values (EWAS) or −log_10_ adjusted *p* values (RNA-Seq). Details of the pathways analysis are in Additional file: Table S7.

Robust linear regression analysis with adjustment for 10 covariates (GA, BW, sex, seven cell-type %) identified 153 BPD-associated CpGs at genome-wide significance level (Bonferroni or BF, *p* < 6.32E−08) and 2871 CpGs at FDR1% (Fig. 4A). More CpGs (76–82%) were hypomethylated in BPD than in nonBPD infants (Additional file 2: Fig. S3A). The 153 BF BPD-CpGs were annotated to a total of 225 nearby genes (Table 3; Additional file 1: Table S5).

**Table 3:** Top 30 CpGs significantly associated with bronchopulmonary dysplasia (BPD) risk on postnatal days.

Postnatal day 14						Postnatal day 28					
CpG	FDR	dMeth	Chr	hg38 position	Associated genes	CpG	FDR	dMeth	Chr	hg38 position	Associated genes
cg05277165	3.71E−09	− 0.077	7	94656502	*PEG10* *SGCE*	cg16039979	9.80E−08	0.044	1	2203919	*LOC105378593* *FAAP20*
cg09655876	2.94E−08	− 0.016	6	161140034	*AGPAT4* *AGPAT4-IT1*	cg19981215	9.80E−08	0.031	11	7222869	*RP11-324J3.1* *MIR302E*
cg00021325	2.94E−08	− 0.050	14	90828068	*LINC02321*	cg26472973	9.80E−08	− 0.0069	7	158140989	*PTPRN2* *LOC105375613*
cg23664662*	1.63E−07	0.093	2	89298788	*IGKV1-37* *LOC107985910*	cg11072045	6.79E−07	− 0.017	1	28368910	*PHACTR4*
cg25273767	1.63E−07	− 0.083	4	151032422	*LRBA*	cg25720930	1.06E−06	− 0.062	19	47728849	*EHD2*
cg25178519	1.63E−07	− 0.031	12	80706526	*LOC105369867* *MYF6*	cg05185749	2.64E−06	0.089	9	114930479	*TNFSF8*
cg06544061	4.86E−07	− 0.056	7	2436788	*CHST12*	cg14002520	4.87E−06	0.034	1	31834669	*SPOCD1*
cg23205276	6.74E−07	− 0.023	1	14943739	*KAZN*	cg23664662*	7.12E−06	0.076	2	89298788	*IGKV1-37* *LOC107985910*
cg18268553	1.13E−06	0.017	1	229420410	*ACTA1*	cg02934888	8.64E−06	− 0.101	3	186458585	*LINC02051* *LINC02052*
cg25391562	1.71E−06	− 0.024	5	90511179	*LYSMD3* *POLR3G*	cg05469706	8.64E−06	− 0.008	7	153411557	*LINC01287*
cg00825168	1.88E−06	− 0.046	1	201496649	*CSRP1*	cg16983486	8.64E−06	− 0.038	11	8832697	*DENND2B*
cg15408512	1.90E−06	0.090	10	133291983	*TUBGCP2* *ADAM8*	cg20363171	1.15E−05	0.025	19	9846868	*PIN1* *OLFM2*
cg05308829	2.86E−06	− 0.036	7	2815991	*GNA12*	cg24523147	2.36E−05	− 0.121	10	100661310	*PAX2*
cg20521882	3.11E−06	− 0.029	11	34175322	*ABTB2* *LOC102723539*	cg21546377	3.53E−05	− 0.027	2	41717608	*RP11-459K11.1* *LOC105374506*
cg04718322	3.32E−06	− 0.055	16	31324491	*ITGAM* *ITGAX*	cg01508428	3.53E−05	− 0.010	20	31587655	*HM13-AS1*
cg17568192	3.35E−06	− 0.066	10	96703744	*PIK3AP1*	cg25830660	3.53E−05	− 0.024	3	197299225	*DLG* *DLG1-AS1*
cg04545015	6.26E−06	− 0.015	1	36100425	*COL8A2* *ADPRS*	cg05031301	3.53E−05	− 0.028	6	144675849	*UTRN*
cg11886100	9.41E−06	− 0.066	17	65244481	*RGS9*	cg16183924	4.92E−05	− 0.033	22	38200455	*MAFF*
cg04912784	9.41E−06	− 0.037	1	166907963	*ILDR2* *MAEL*	cg01872077	6.08E−05	0.018	2	218781649	*CYP27A1*
cg23077884	9.41E−06	− 0.067	2	156539323	*GPD2*	cg03565499	6.08E−05	− 0.006	6	89658044	*MDN1-AS MDN1* *LYRM2*
cg03881544	1.14E−05	0.018	4	5710854	*EVC* *EVC2*	cg21387752	6.08E−05	− 0.011	17	48602926	*HOXB HOXB6* *HOXB-AS3*
cg22993554	1.15E−05	0.026	8	18840540	*PSD3*	cg19165344	6.08E−05	− 0.037	22	29389366	*RFPL1* *AP1B1*
cg14761552	1.17E−05	− 0.006	2	190880676	*GLS*	cg00664416	6.18E−05	− 0.052	1	37795520	*MANEAL*
cg20610177	1.29E−05	− 0.034	16	482468	*RAB11FIP3* *LOC105369937*	cg03278250	6.75E−05	0.003	22	43400193	*LINC01639*
cg14634053	1.29E−05	− 0.037	12	101660148	*MYBPC1 LOC105369937*	cg03263117	7.22E−05	0.006	17	47721401	*TBX21*
cg14933812	1.69E−05	− 0.050	1	25813614	*SELENON* *MTFR1L*	cg19221542	8.86E−05	0.002	12	130160084	*FZD10* *FZD10-AS1*
cg03717503	1.70E−05	− 0.014	16	86700570	*RP11-58A18.2* *LINC02189*	cg14039306	9.35E−05	− 0.027	4	4860513	*MSX1*
cg03442269	2.94E−05	− 0.107	2	30959195	*GALNT14*	cg08368948	9.91E−05	− 0.003	12	52655926	*KRT2*
cg19741032	3.00E−05	0.161	13	114313349	*CHAMP1*	cg19977004	9.91E−05	− 0.037	11	1461333	*LOC105376514* *BRSK2*
cg05762432	3.25E−05	− 0.125	4	168741927	*PALLD*	cg05750078	1.5E−04	− 0.031	10	1525551	*ADARB2* *ADARB2-AS1*

Selected from a total of 153 CpGs (225 gene annotated) or 116 CpGs (172 genes annotated) significantly different on postnatal day 14 (*p* < 6.05E−08) or day 28 (*p* < 6.52E−08), respectively, with Bonferroni correction between BPD or nonBPD treated with any day(s) of oxygen (nonBPD-AnyO_2_). Robust linear regression adjusted with 10 covariates (gestational age, sex, birth weight, 7 cell-type %). *dMeth* methylation difference in BPD relative to nonBPD-AnyO_2_, *Chr* chromosome, *hg38* human genome assembly GRCh38. Full list of the differentially methylated CPGs at false discovery rate 1% cut-off is in Additional file: Table S5 (day 14; 2871 CpGs annotated to 3801 genes), S8 (day 28; 2299 CpGs annotated to 3173 genes). *Overlap between two postnatal days.

DESeq2 analysis with adjustment for nine covariates (GA, BW, sex, cell-type % of CD4 + T, CD8 + T, B cell, NK, granulocyte, and monocyte) determined DEGs (731 genes at *p* < 0.01) in peripheral blood RNA of BPD samples on day 14 (Table 4; Additional file 1: Table S6). Hierarchical clustering analysis of 731 DEGs demonstrated more genes (453/731) were overexpressed in BPD than in nonBPD infants (Fig. 4B; Additional file 2: Fig. S3A,B), which suggested potential demethylation-mediated transcriptional activation.

**Table 4:** Representative differentially expressed genes (DEGs) in peripheral blood cells of preterm infants with bronchopulmonary dysplasia (BPD).

Postnatal Day	Gene	FDR	*p*	FC	Description
Day 14	*NAAA*	1.13E−02	9.19E−07	− 1.74	N-acylethanolamine acid amidase
	*PPM1N*	2.88E−02	6.47E−06	3.35	Protein phosphatase, Mg2 + /Mn2 + dependent 1N
	*H2AC14*	2.88E−02	1.14E−05	3.10	H2A clustered histone 14
	TNFSF8*	2.88E−02	1.28E−05	− 1.74	TNF superfamily member 8
	*CCNA2*	2.88E−02	1.63E−05	2.45	Cyclin A2
	*RRM2*	2.88E−02	1.73E−05	2.47	Ribonucleotide reductase regulatory subunit M2
	*WLS*	2.88E−02	1.87E−05	− 3.39	Wnt ligand secretion mediator
	*PATJ*	3.16E−02	2.31E−05	− 1.89	PATJ crumbs cell polarity complex component
	FLT1*	3.56E−02	2.89E−05	− 1.82	Fms related receptor tyrosine kinase 1
	*RSAD2*	3.68E−02	3.97E−05	6.91	Radical S-adenosyl methionine domain containing 2
	DBF4B*	3.68E−02	4.12E−05	1.91	DBF4 zinc finger B
	*TUBG1*	3.68E−02	4.30E−05	1.96	Tubulin gamma 1
	*CLSPN*	3.68E−02	4.93E−05	2.29	Claspin
	*FAM114A2*	3.68E−02	5.20E−05	1.99	Family with sequence similarity 114 member A2
	*ZNF155*	3.68E−02	5.50E−05	− 1.73	Zinc finger protein 155
	*POM121*	3.68E−02	5.62E−05	1.91	POM121 transmembrane nucleoporin
	*FOXM1*	3.68E−02	5.80E−05	2.61	Forkhead box M1
	*CDCA2*	3.68E−02	5.98E−05	2.39	Cell division cycle associated 2
	*ENC1*	4.11E−02	7.00E−05	− 1.83	Ectodermal-neural cortex 1
	*SPC25*	4.39E−02	7.85E−05	3.80	SPC25 component of NDC80 kinetochore complex
	*HBG1*	8.28E−02	5.78E−04	39.62	Hemoglobin subunit gamma 1
	CD28*	8.62E−02	8.59E−04	− 1.74	CD28 molecule
	*IFI6*	9.41E−02	1.40E−03	4.71	Interferon alpha inducible protein 6
	*IL15RA*	9.41E−02	1.39E−03	2.15	Interleukin 15 receptor subunit alpha
	*IFIT1*	1.07E−01	2.36E−03	6.89	Interferon induced protein with tetratricopeptide repeats 1
	*AURKB*	1.11E−01	2.99E−03	1.95	Aurora kinase B
	SLC18A2*	1.11E−01	3.04E−03	− 2.22	Solute carrier family 18 member A2
Day 28	GNLY*	1.17E−02	8.61E−07	3.81	Granulysin
	*MCM6*	1.00E+00	2.10E−04	− 1.75	Minichromosome maintenance complex component 6
	*TRDC*	1.00E+00	1.07E−03	1.99	T cell receptor delta constant
	*GRIP1*	1.00E+00	1.14E−03	2.31	Glutamate receptor interacting protein 1
	*LTF*	1.00E+00	1.65E−03	− 6.90	Lactotransferrin
	*HEBP1*	1.00E+00	1.69E−03	3.16	Heme binding protein 1
	*PLA2G4A*	1.00E+00	2.39E−03	− 1.81	Phospholipase A2 group IVA
	*TCF19*	1.00E+00	2.73E−03	− 1.96	Transcription factor 19
	*PTPN11*	1.00E+00	3.00E−03	− 1.40	Protein tyrosine phosphatase non-receptor type 11
	*KNDC1*	1.00E+00	3.30E−03	2.41	Kinase non-catalytic C-lobe domain containing 1
	*MPO*	1.00E+00	3.58E−03	− 4.32	Myeloperoxidase
	*ANKRD16*	1.00E+00	3.82E−03	1.68	Ankyrin repeat domain 16
	*CEACAM21*	1.00E+00	3.85E−03	− 1.75	CEA cell adhesion molecule 21
	*SNORD89*	1.00E+00	4.29E−03	2.34	Small nucleolar RNA, C/D box 89
	*CDC6*	1.00E+00	4.41E−03	− 2.22	Cell division cycle 6
	*CHEK1*	1.00E+00	5.34E−03	− 1.83	Checkpoint kinase 1
	*JUNB*	1.00E+00	1.40E−02	1.71	JunB proto-oncogene
	*PRTN3*	1.00E+00	1.43E−02	− 4.15	Proteinase 3
	*IL23A*	1.00E+00	2.33E−02	1.67	Interleukin 23 subunit alpha
	*RAG1*	1.00E+00	3.05E−02	− 2.37	Recombination activating 1
	RCOR1*	1.00E+00	4.48E−02	− 1.40	REST corepressor 1

A total of 731 DEGs (*p* < 0.01or 312 DEGs (*p* < 0.05), 1 DEG at FDR 10%) significantly varied between BPD and nonBPD-exposed to any day(s) of oxygen (O_2_) on postnatal days 14 or 28, respectively, determined by RNA-sequencing analysis. *FC* expression difference in BPD vs. nonBPD-AnyO_2_. Full lists of the DEGs in Additional file: Tables S6 and S10. *Genes overlapped with those annotated to epigenome-wide association study of the corresponding time.

Differentially methylated CpG-annotated genes were mainly enriched in organ and cellular development and morphology signaling (e.g., *GNA12*, *NCOA1*, *PDGFA*, *MYH10*), telomere length and cellular senescence (e.g., *CDK6*, *TERT*, *TNKS*), neutrophilic inflammation and immune responses (e.g., *ADAM8*, *CD55*, *IFNA1*, *ITGAM*, *MMP8*, *PIN1*) and oxidative stress (e.g., *ALDH3B1*, *GGT1*) (Fig. 4C; Additional file 1: Table S7). Transcriptome changes in infants who developed BPD included genes involved in chromosome segregation and DNA repair (e.g., *AURKB*, *BRCA2*, *CCNA2*, *H4C13*) potentially inhibiting cell cycle progression and directing cells toward apoptosis or DNA damage repair (Fig. 4C; Additional file 1: Table S7). In BPD infants, there may be decreased proliferation of lymphocytes and reduced T cell-mediated immune responses (e.g., *CD2*, *IL15RA*, *JAK1*, *TNFSF8*). In addition, enrichment in pathways for IFN signaling and cytokine ‘storm’ (e.g., *IFI6*, *IFITM1*, *IL1RN*, *MYD88*, *LCK*), antioxidant response regulated by nuclear factor erythroid 2-related factor 2/glutathione redox (e.g., *CHST12*, *GCLM*, *GSR*, *NFE2L2*, *SOD1*), and mitochondrial dysfunction (e.g., *ACO1*, *COX7B*, *NDUFS4*) indicated severe oxidative stress and inflammation in infants who developed BPD. The group of genes that overlapped between epigenome and transcriptome on day 14 (85 genes with FDR 1% epigenome changes, Table S7) showed enrichment in altered T cell development, T cell-mediated immune responses, and inflammation/oxidative stress-mediated cellular senescence (Additional file 2: Fig. S3C). These pathway analysis results were consistent with the reduced lymphocyte percentage, increased granulocyte percentage and the increased NLR in BPD infants on day 14 (shown in Fig. 2A,B).

### BPD-associated CpGs and DEGs in peripheral blood cells on postnatal day 28

**Figure 5:**
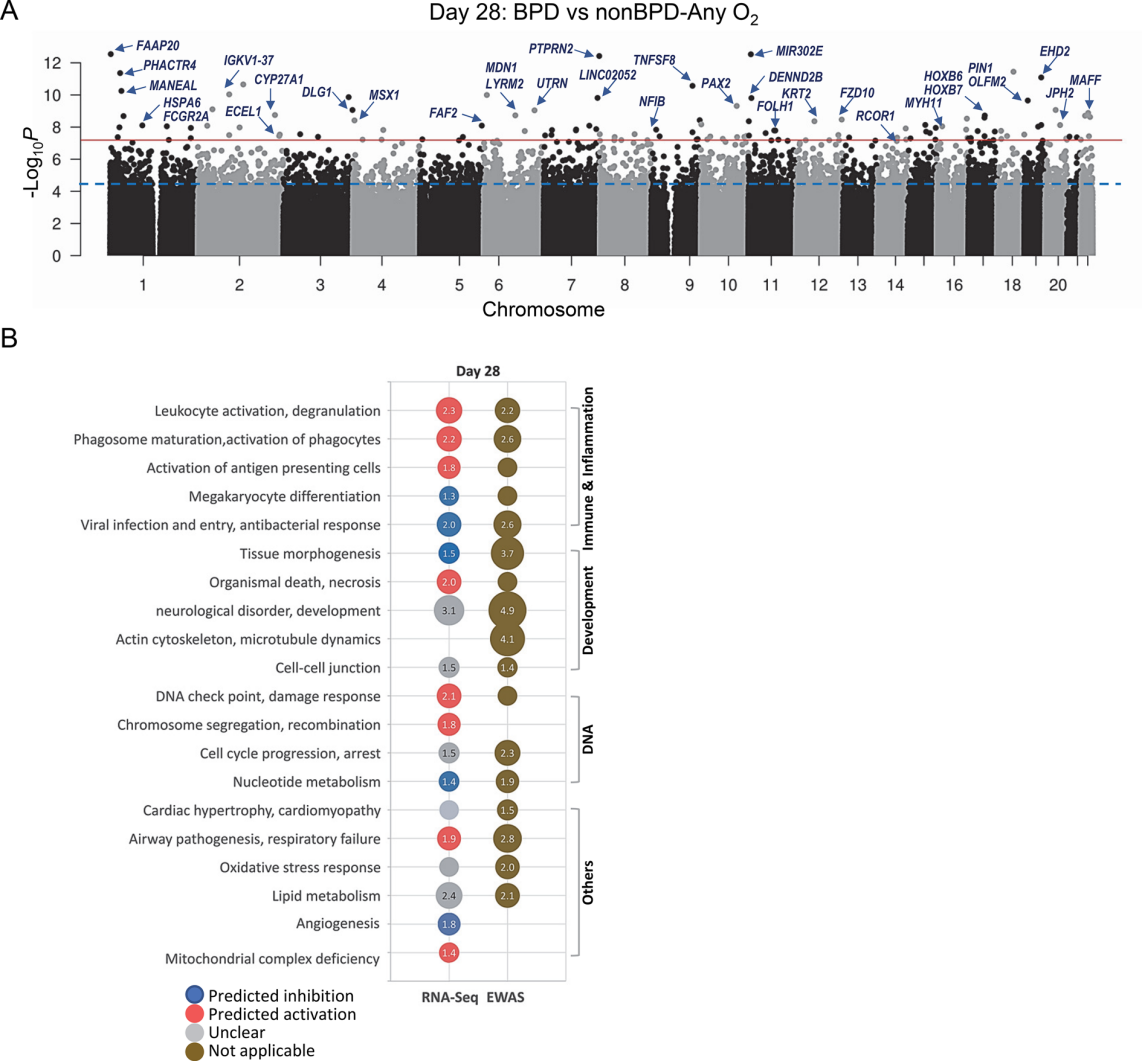
Epigenome-wide association study (EWAS) and genome-wide gene expression analysis for bronchopulmonary dysplasia (BPD) development in blood cells from premature infants on postnatal day 28. (**A**) A Manhattan plot displays BPD-associated CpGs on day 28 by comparison of BPD (n = 11) and nonBPD (n = 48, ≥ 1 day of oxygen (O_2_) treated in the newborn intensive care unit) with adjustment for covariates (seven cell types, sex, gestational age, birth weight). Robust linear regression model elucidated 116 CpGs with Bonferroni correction (*p* < 6.46−E08, red line) and 2299 CpGs at FDR 1% (*p* < 3.0E−05, blue line). (**B**) Top-ranked pathways and gene ontologies of BPD-associated genes and BPD CpG-annotated genes determined by pathway analysis tools depict similar or unique molecular events in transcriptome and epigenome. Circle size and label indicate −log_10_ *p* values (adjusted *p* for RNA-Seq). Details of the pathways are in Additional file: Table S9.

BPD diagnosis was associated with 116 CpGs at BF (2299 CpGs at FDR 1% cut-off) on day 28 (Fig. 5A; Table 3; Additional file 1: Table S8) and DNA hypomethylation (demethylation of ~ 80% of CpGs) was evident in infants who developed BPD, similarly to the result on day 14 (Additional file 2: Fig. S4A). Pathways of the BPD-CpG-annotated genes were similar to those seen on day 14 and tissue morphology, neuron and body development, and cellular assembly (e.g., *CACNB2*, *CHD7*, *FGF8*, *HOXB6*, *PAX2*) were noted (Fig. 5B; Additional file 1: Table S9). In addition, BPD-CpG annotated genes observed on day 28 (Fig. 5B) were involved in immune and inflammatory events, including activation of immune and inflammatory cells, phagosome formation, infection, and platelet development (e.g., *FCGR2A*, *GPR55*, *IGKV1-37*, *NFIB*, *TNFSF8*), nucleotide metabolism (e.g., *ADARB2*, *NME6*), cholesterol metabolism (e.g., *CYP27A1*, *MBTBS1*), and oxidative stress (e.g., *ALOX12*, *MAFF*, *NXN*).

BPD-DEGs on day 28 included 312 genes (*p* < 0.05) (Table 4; Additional file 1: Table S10; Additional file 2: Fig. S4B). Enriched pathways of DEGs on day 28 were similar to those on day 14 such as chromosome segregation and DNA damage (e.g., *BLM*, *CHEK1*), activation of phagocytosis and degranulation (e.g., *GNLY*, *MPO*, *LTF*), leukocyte activation and immune response (e.g., *IL23A*, *PIK3R3*, *RAG1*, *TRDC*), neurological disorder (e.g., *ATP6V1A*, *DUSP*), and lipid metabolism (e.g., *ACACA*, *SCD*) (Fig. 5B; Additional file 1: Table S9). The epigenome-transcriptome common 30 genes on day 28 were also enriched in these pathways (Additional file 2: Fig. S4C), indicating the transcriptome changes are parallel with epigenome changes on day 28 in infants who are developing BPD. Overall, this indicated that altered leukocyte activation and antimicrobial response, DNA damage and nucleotide metabolism, lipid metabolism, and angiogenesis and other growth-related events continue on day 28 in premature infants who went on to develop BPD.

### NICU O_2_ therapy-associated CpGs and DEGs in peripheral blood cells on postnatal day 14

**Figure 6:**
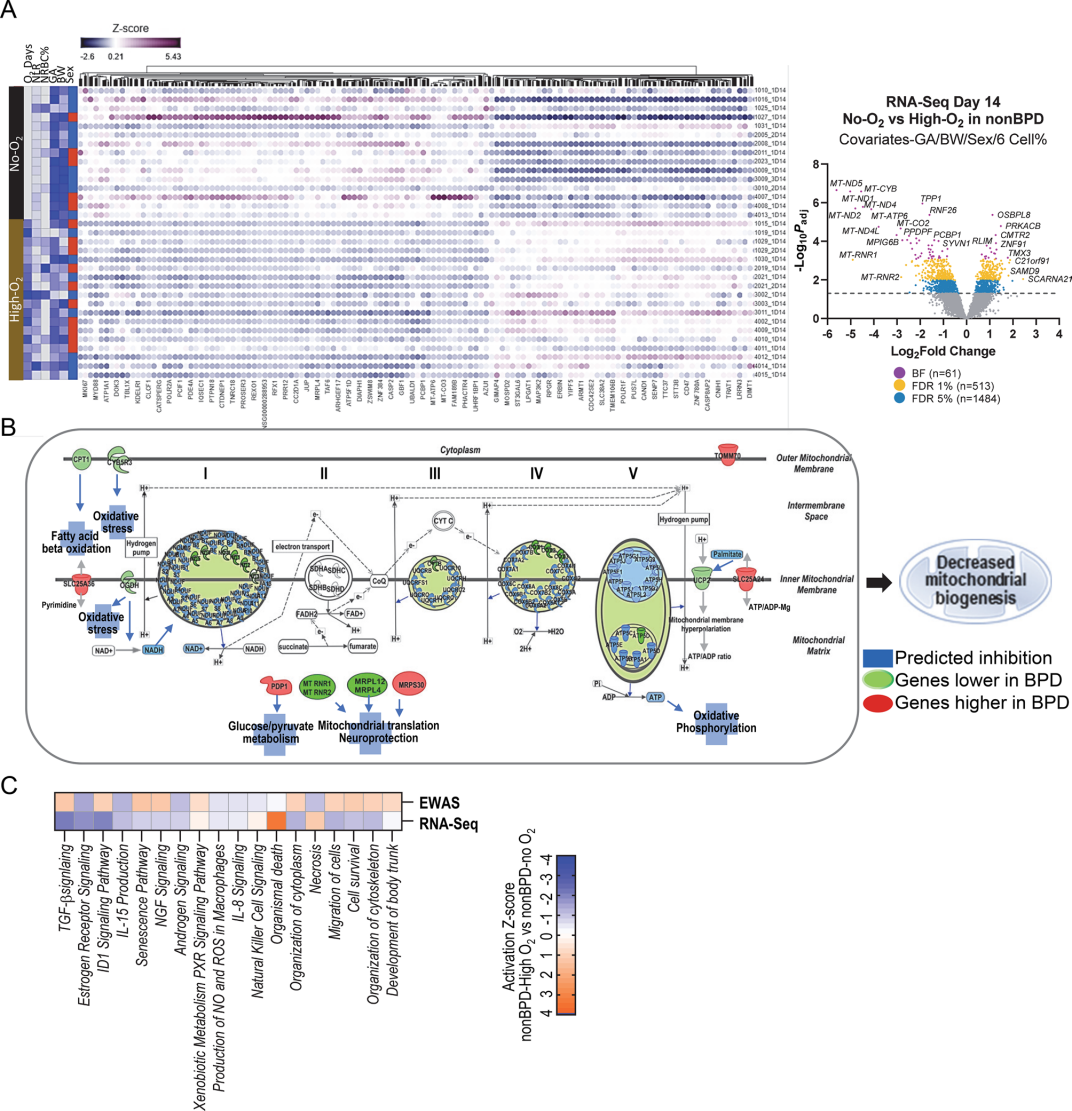
Genome-wide gene expression analysis of oxygen (O_2_) treatment in neonatal intensive care unit (NICU) and comparison with epigenome-wide association study (EWAS) in preterm infants on postnatal day 14. (**A**) Heat map of O_2_-differentially expressed genes (n = 513 at FDR 1% determined by RNA sequencing (RNA-Seq) between nonBPD infants exposed to prolonged O_2_ (≥ 14 days, High-O_2_, n = 18) and those to no O_2_ exposure (No-O_2,_ n = 15) with adjustment for nine covariates [six cell types, sex, gestational age (GA), birth weight (BW))]. Down-regulated and up-regulated genes in High-O_2_ relative to No-O_2_ are in orange and blue, respectively. NICU O_2_ days, neutrophil–lymphocyte ratio (NLR), nucleated red blood cell (NRBC) %, GA, and BW (color intensity = scale) as well as sex (Red-Male, Blue-Female) are labeled for each sample. Samples by row and genes by column. A volcano plot depicts Log_10_-transformed adjusted *p* values against Log2-transformed fold changes of differentially expressed genes by prolonged O_2_-exposure in nonBPD. Heatmaps, volcano plot and diagrams created using Partek Flow (Partek Inc., Chesterfield, MO; https://www.partek.com/partek-flow/) (**B**) Pathway analysis of differentially expressed genes predicted that NICU O_2_ exposure disturbs various molecular events including mitochondrial oxidative phosphorylation and biogenesis, which leads to mitochondrial dysfunction and related diseases such as neurological disorders. (**C**) Comparison of epigenome and transcriptome changed by ≥ 14 days of NICU O_2_ supplementation in premature infants elucidates similar enriched pathways and gene ontologies such as developmental signaling (TGF-β, NGF, ID-1), cellular assembly (cytoskeleton, cytoplasm), reactive oxygen species (ROS) production and xenobiotic metabolism, inflammation (interleukin 8, IL-8), and T cell differentiation (IL-15). The activation z-scores of methylome changes (EWAS) are in general reciprocal to expression changes (RNA-Seq).

To assess the epigenetic and transcriptomic response to NICU O_2_ supplementation in prematurity without regard to disease processes, we examined these endpoints on postnatal day 14 in nonBPD infants exposed to O_2_ for ≥ 14 days (High-O_2_), and compared them to nonBPD infants who were never exposed to O_2_ (No-O_2_). EWAS (n = 20 for High-O_2_, n = 28 for No-O_2_) determined 346 differentially methylated loci at FDR 1%, which varied up to 7.5% methylation difference and were annotated to 529 genes (Table 5; Additional file 1: Table S11; Additional file 2: Fig. S5A). RNA-Seq (n = 18 for High-O_2_, n = 15 for No-O_2_) determined 513 DEGs at FDR 1% (1480 DEGs at FDR 5%) with the greatest fold changes observed in mitochondria-encoded mitochondrial genes (Table 5; Additional file 1: Table S12). About 61% of DEGs (314/513) were markedly downregulated by O_2_ exposure (Fig. 6A). High-O_2_ infants had relatively higher NLR (Fig. 6A) and smaller GA and BW compared to No-O_2_ infants. In addition, more males (10/15, 67%) required prolonged O_2_ treatment than females (8/18, 44%).

**Table 5:** Representative differentially methylated CpGs and differentially expressed genes (DEGs) associated with prolonged oxygen (O_2_) exposure on postnatal day 14.

CpG	FDR	dMeth	Chr	hg38 position	Associated genes
cg05355320	8.59E−06	0.042531	19	55059986	*RDH13*
cg10315128	1.31E−05	0.004523	18	51030261	*LOC105372117 SMAD4*
cg01772192	2.97E−05	− 0.0629	15	80977761	*MESD*
cg08975094	5.41E−05	− 0.00325	16	89922880	*MC1R TUBB3*
cg01071808	6.09E−05	0.031849	21	10521563	*TPTE BAGE2*
cg24525563	6.76E−05	0.028264	7	32961715	*FKBP9*
cg15612682	8.22E−05	− 0.0032	12	123602356	*TMED2 DDX55*
cg17172683	8.22E−05	0.048487	2	132586045	*GPR39 LOC105373623*
cg01917080	8.61E−05	− 0.04708	12	1663338	*MIR3649*
cg02256969	1.41E−04	− 0.01706	16	2858244	*PRSS22*
cg15152208	1.47E−04	− 0.03727	21	32475600	*EVA1C*
cg15893057	2.70E−04	0.003187	4	140282044	*SCOC-AS1 SCOC*
cg17306740	3.36E−04	0.02259	20	57620378	*ZBP1*
cg04428115	3.36E−04	− 0.02107	12	131002449	*ADGRD1*
cg00171275	3.36E−04	0.01405	18	59909068	*PMAIP1*
cg16951654	3.36E−04	− 0.00452	6	32839564	*PSMB8 TAP2 PSMB8-AS1*
cg10192836	3.36E−04	− 0.00615	1	19882143	*LOC105376823 OTUD3*
cg08601917	3.36E−04	0.007117	16	66604493	*CMTM4 CMTM3*
cg24831541	3.36E−04	0.024357	17	4709750	*ARRB2 PELP1-DT*
cg25922935	3.47E−04	− 0.05769	3	157029702	*LEKR1 LINC02029*
cg23220346	3.61E−04	0.007405	3	35744538	*ARPP21 MIR128-2*
cg17849972	4.19E−04	0.011417	1	203085848	*MYOPARR MYOG*

Gene	FDR	FC	Description		
*MT-ND5*	2.23E−07	− 48.76	NADH dehydrogenase subunit 5		
*MT-CYB*	2.63E−07	− 23.50	Cytochrome b		
*MT-ND1*	2.63E−07	− 32.56	NADH dehydrogenase subunit 1		
*TPP1*	1.09E−06	− 3.72	Tripeptidyl peptidase 1		
*MT-ND4*	1.66E−06	− 22.21	NADH dehydrogenase subunit 4		
*MT-ND2*	2.00E−06	− 27.95	NADH dehydrogenase subunit 2		
*MT-ATP6*	2.17E−06	− 17.26	ATP synthase F0 subunit 6		
*RNF26*	4.22E−06	− 3.03	Ring finger protein 26		
*OSBPL8*	4.22E−06	2.17	Oxysterol binding protein like 8		
*PRKACB*	1.62E−05	2.79	Protein kinase cAMP-activated catalytic subunit beta		
*MT-ND4L*	1.76E−05	− 13.85	NADH dehydrogenase subunit 4L		
*MT-CO2*	2.21E−05	− 7.15	Cytochrome c oxidase subunit II		
MPIG6B*	4.78E−05	− 8.13	Megakaryocyte and platelet inhibitory receptor G6b		
*CMTR2*	4.78E−05	2.40	Cap methyltransferase 2		
*PPDPF*	5.27E−05	− 5.59	Pancreatic progenitor cell differentiation and proliferation factor		
*FAM189B*	7.16E−05	− 4.51	Family with sequence similarity 189 member B		
*ZNF385A*	8.45E−05	− 5.91	Zinc finger protein 385A		
*PCBP1*	8.62E−05	− 2.62	Poly(rC) binding protein 1		
*MT-ND3*	8.62E−05	− 6.82	NADH dehydrogenase subunit 3		
*CTDNEP1*	9.08E−05	− 4.11	CTD nuclear envelope phosphatase 1		
*SYVN1*	9.60E−05	− 2.31	Synoviolin 1		
*ZNF91*	1.25E−04	2.41	Zinc finger protein 91		
*GP1BA*	1.26E−04	− 5.31	Glycoprotein Ib platelet subunit alpha		
*MT-CO1*	1.26E−04	− 4.45	Cytochrome c oxidase subunit I		
*PLBD2*	1.63E−04	− 3.11	Phospholipase B domain containing 2		

Differentially methylated CpGs (346 at false discovery rate (FDR) 1% cut-off) and DEGs (513 genes at FDR 1%, 1480 genes at FDR 5%) between nonBPD-exposed to prolonged O_2_ (≥ 14 days, High-O_2_) in the neonatal intensive unit and nonBPD-no O_2_ (No-O_2_) exposure. *dMeth* methylation difference in ≥ 14 days-O_2_ vs. No-O_2_. *hg38* human genome assembly GRCh38, *FC* expression difference in High-O_2_ vs No-O_2_. Robust linear regression adjusted with 10 covariates (gestational age, sex, birth weight, 7 cell-type %) to determine differentially methylated CpGs and 9 covariates (gestational age, sex, birth weight, 6 cell-type %) to determine DEGs. *Gene overlapped with CpG-annotated genes. Full lists of data in Additional file: Tables S11 and S12.

Transcriptome differences in the blood of O_2_ supplemented neonates strongly pointed to the inhibition of mitochondrial function and morphology including oxidative phosphorylation (e.g., *MT-ATP6*, *MT-CO2*, *MT-ND5*, *MT-CYB*, *LRPPRC*) (Fig. 6B). O_2_-dependent DEGs were also involved in neuronal development and mental retardation (e.g. *CREBBP*, *ROCK1*, *CUL3*), growth retardation (e.g. *HHEX*, *MT-ND4*, *NCOA6*, *NCOR2*), lymphopoiesis (e.g. *BCOR*, *GPR183*, *RASGRP4*), neutrophilic inflammation (e.g., *MOSPD2*, *DOK3*, *OSCAR*), and DNA damage and repair (e.g., *BAX*, *BRD4*, *LIG1)* (Additional file 2: Fig. S5B). O_2_-altered methylome was predicted to affect cellular organization, neuron and organ development (e.g., *MME*, *NCOR1*, *NGF*, *PDLIM7*, *RPL24*, *TRIO*), and lymphoid organ and lymphocyte differentiation (e.g., *HLA-DQB1*, *RAG1*, *TYK2*) (Additional file 2: Fig. S5B).

Comparison of epigenomic and transcriptomic pathways influenced by O_2_ supplementation showed similarities in neuron and tissue development signaling pathways (e.g., TGF-β, NGF, ID-1), cellular assembly, xenobiotic metabolism, cellular senescence, reactive oxygen species production, cell and organ death, and inflammation and infection (Fig. 6C). The pathway activation z-scores of the methylation changes were, in general, inversely related to those of gene expression changes in these functions.

## Discussion

**Figure 7:**
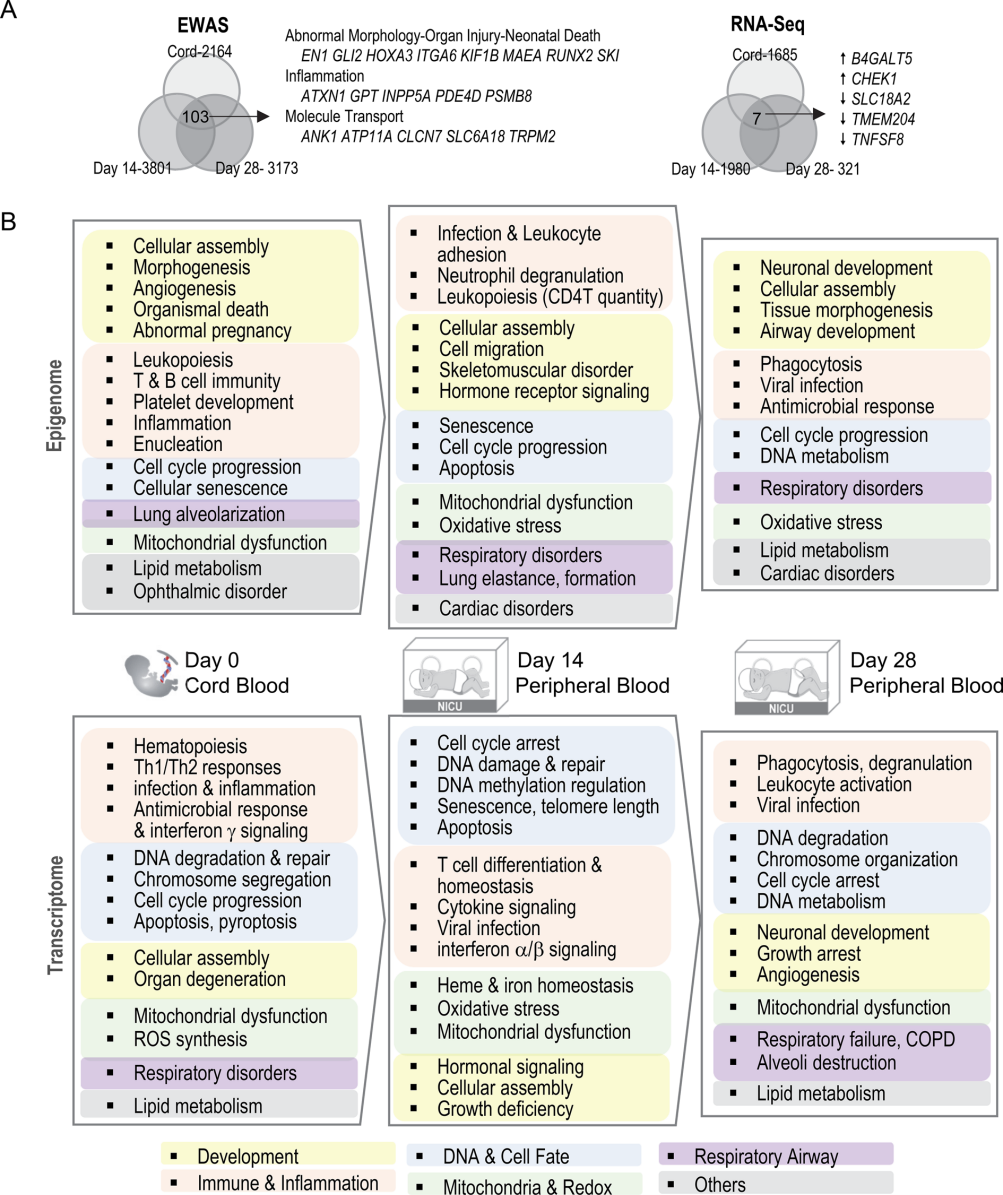
Comparison of epigenome-wide association study (EWAS) and genome-wide gene expression analysis of bronchopulmonary dysplasia (BPD) risk in cord blood and postnatal day peripheral blood. (**A**) Overlapping genes annotated to differentially methylated CpGs determined by EWAS or differentially expressed genes determined by RNA-sequencing (RNA-Seq) between different times of samples. Common 103 genes from extended list (false discovery rate 1%) from EWAS were mainly associated with organ morphology and neonatal death, inflammation, and molecular transport. RNA-Seq elucidated seven overlapping genes (at *p* < 0.05) increased (↑) or decreased (↓) in BPD compared to nonBPD. (**B**) BPD-associated pathways and gene ontologies predicted from epigenome and transcriptome changes were compared by time.

This study analyzed DNA methylation and gene expression profiles of cord and venous blood during the 1st month after premature birth and reported on numerous molecular and cellular differences observed in neonates later diagnosed with BPD (summary in Fig. 7). We observed that GA was significantly correlated with decreased proportions of lymphocytes and increased proportions of granulocytes in both cord and peripheral blood. These changes were also reflected in the NLR, particularly in the extremely preterm neonates, including the infants who ultimately developed BPD. NLR is easily assessed by standard blood count methods and the NLR in the first weeks of life could indicate babies at high risk for later developing BPD as has been suggested^27^. The present findings are consistent with this notion as we observed greater NLR in BPD babies at each of the time points studied.

The NLR is likely to be driven by inflammation, and both systemic and lung inflammation by antenatal infection, chorioamnionitis (e.g., rubella, *Ureaplasma* species), and postnatal sepsis (e.g., *E. coli*, group B streptococcus) have been reported to increase BPD risk^33–35^. The NLR and the pathway enrichment in: interferon signaling (cord blood and day 14 methylome and transcriptome); neutrophil degranulation (day 14 methylome, day 28 transcriptome), hyper-cytokinemia (day 14 transcriptome); complement system activation (day 14 methylome); and phagocytosis (day 28 methylome and transcriptome), collectively suggested microbial infection, neutrophilic inflammation, and altered innate immune responses in infants who developed BPD. Key genes in the innate immune response that were differentially expressed or methylated included *GNLY* and granzyme (*GZMA*), the antimicrobial peptides from killer lymphocyte granules causing microptosis^36^. In addition, antimicrobial lactoferrin (*LTF*), neutrophilic myeloperoxidase (*MPO*), anti-streptococcus *AGER* and cathepsin D (*CTSD*), interferon alpha 1 (*IFNA1*), interferon induced transmembrane proteins (*IFITM1*, *IFITM3*), and the negative regulator of interferon response (*NPIR*) are likely to be important players in innate immune modulation of BPD pathogenesis. This is consistent with our observation of a higher frequency of infection in the BPD infants (Table 1), including sepsis and pneumonia (Additional file 1: Table S1), and also with an adaptive immune response as indicated by apparent shifts from naïve to memory among B cells, CD4 T cells and CD8 T cells (Additional file 1: Table S2).

Consistent with the lowered percentages of lymphocytes (CD4 + T, CD8 + T, B cells) in BPD compared to nonBPD infants, EWAS and RNA-seq coordinately suggested suppressed T cell immunity in BPD infants (Fig. 7). Mucosal immune imbalance due to lymphocyte insufficiency may mediate respiratory infection and inflammation, and play a role in lung damage and aberrant development. O_2_-induced DNA damage and cell cycle arrest during the neonatal period (Figs. 4C; 5B) may also contribute to decreased lymphopoiesis in BPD. Immune cell senescence due to oxidative stress and telomere shortening was also suggested by telomere-related epigenetic (e.g., *TERT*, *TNKS*) or transcriptomic (e.g., *PINX1*, *TERF2*) changes in BPD cases on day 14. In cord blood, epigenetic inhibition of IL-2 may affect downstream transcriptomics leading to improper lymphocyte differentiation and activation in BPD (Fig. 3B). IL-2 from CD4 + T and CD8 + T cells is a powerful modulator of T cell growth, leukocyte activation, and IL-18 stimulation of IFN production and NK cell cytotoxicity^29^. Downregulation of immune surface marker genes related to T cell maintenance and T and B cell activation (e.g., CD28, CD27, CD7, CD79B, CD83) in cord and/or peripheral blood further supports skewed B and T cell immunity in preterm infants who developed BPD. In addition, decreased expression of tumor necrosis factor superfamily member 8 (*TNFSF8*) encoding CD153, one of the 7 BPD-DEGs in common to all timepoints, suggested interruption of T cell-dependent anti-mycobacterial immune response^37^ and also class switch DNA recombination and immunoglobulin production^38^ in BPD pathogenesis. T cell inadequacy has been suggested as an immunological clue for BPD prediction. For example, a downregulation of T cell receptor signaling was reported in postnatal blood (5–28 days) from preterm BPD babies^14,19^. Scheible et al.^39^ reported that premature infants had a lowered level of CD31 + CD4 + T cells on discharge and showed increased risk for respiratory complications at 1 year of age compared to full-term babies^37,38^.

In addition to the immunological biomarkers, we also observed development- and alveolarization-related epigenetic and transcriptomic markers in cord blood of infants who developed BPD^30,31,40^. Concurrently with BPD epigenome data^22^, we identified *SPOCK2* and *AGER* among the BPD-DEGs in cord blood. *SPOCK2*, a BPD susceptibility gene identified from GWAS^7,30,31^, was reported to be deleterious in BPD development as its overexpression in the lung altered neonatal mouse lung alveolarization and exacerbated hyperoxia-caused alveolar simplification while anti-SPOCK2 antibody treatment in mice improved it^7,30^. AGER is a type 1 alveolar cell differentiation marker^31^ and prenatal exposure to nicotine in mice arrested alveolarization and upregulated the AGER signaling pathway^41^. In addition, *Ager* knock-in mice were generated to study alveolar development and type 1 cell turnover during injury and repair^42^.

A unique aspect of this study was the opportunity to examine the in vivo response of blood cells to O_2_ therapy among nonBPD infants. Comparing preterm infants who received at least 14 days of NICU O_2_ supplementation with those who never received O_2_ therapy, we observed a dramatic downregulation of a large set of mitochondria-related genes, either mitochondrial DNA-encoded (e.g., *MT-ND5*, *MT-CYB*, *MT-CO2*, *MT-ATP6*) or nuclear DNA-encoded (e.g., *CPT1A*, *LRPPRC*, *SLC25A24*, *UCP2*). Relative to those who never received O_2_ therapy, this down-regulation also occurred in BPD neonates. These genes are involved in mitochondrial DNA-related diseases such as mitochondrial leukoencephalopathy, mitochondrial myopathy, ventricular preexcitation, optic and retinal disorders, and type I diabetes (Additional file 2: Fig. S5B). Other downregulated genes (e.g., *BAX*, *HTT*, *IGF1R*, *OGDH*, *TGFB1*) have also been associated with mitochondrial morphology and permeability. Consistent with our observation, Das et al.^43^ reported that hyperoxia exposure reduced mitochondrial oxidative phosphorylation complex (I and II) activity in lung epithelial cells. In addition, inhibition of mitochondrial respiration caused arrested alveolarization of neonatal mice^44^, indicating mitochondrial biogenesis is critical in lung maturation. In addition, it has been reported that vascular endothelial mitochondrial function is associated with BPD^45^. We observed mitochondrial gene dysregulation and predicted functional abnormality in blood cells at day 14 of O_2_ therapy in both BPD and nonBPD neonates relative to neonates with no O_2_ therapy. While down-regulated mitochondrial biogenesis was observed among neonates with O_2_ therapy and may be caused by this treatment, it is possible the mitochondrial effects are reflecting the infant’s poor blood O_2_ saturation in the neonatal period, rather than a response to treatment. Overall, mitochondrial dysfunction may be one of the underlying mechanisms driving BPD in susceptible infants. However, while nonBPD neonates are able to respond and recover from this exposure, the neonates who develop BPD continue to need O_2_ therapy for many weeks to maintain optimal blood O_2_ saturation levels. It suggests that additional susceptibility factors such as genetic influences or prenatal conditions that lead to a deficit in the response to O_2_ therapy are likely to be involved^45^.

Optimal O_2_ supplementation in the NICU is essential to reduce the risk of respiratory mortality and other morbidities. However, in addition to BPD, preterm babies who require prolonged O_2_ treatment often develop neurological disorders, such as cognitive deficits in the absence of apparent brain injury, and are at higher risk of morbidities including retinopathy of prematurity (ROP) and cardiac disorders^46,47^. Mitochondrial dysfunction due to oxidative stress is associated with adult neurodegenerative disorders^48^ and cardiovascular toxicity^49^, and has been proposed as a mechanism in preterm morbidities^50^. Our observation of O_2_ treatment-induced mitochondrial effects in blood cells supports this idea.

## Conclusions

The small size of the examined cohort and the low incidence of BPD in this study limited the statistical power for many comparisons and therefore, any conclusions should be interpreted with caution. However, despite these limitations, epigenomic and transcriptomic profiling of blood from preterm infants receiving O_2_ supplementation revealed time-dependent immunopathological events in the early weeks of life before they were diagnosed with BPD. Altered methylation and dysregulated transcription presumably have affected neutrophil and lymphocyte proportions, T cell and adaptive immune responses, inflammation and phagocytosis, cellular assembly, and repair of DNA damage in premature neonates. Prolonged O_2_ treatment highly suppressed mitochondrial gene expression, which may be driving lung pathology and be implicated in a variety of neuronal and optical disorders of prematurity. These molecular and cellular outcomes such as NLR may be predictors of BPD susceptibility and should be followed up in larger studies of BPD. Similarity of pathways and overlapping genes between two genomic networks suggested interplay between epigenetics and transcriptomics in BPD pathogenesis. These results provided insights into mechanisms of BPD and warrant further investigation into the clinical relevance of blood methylomic and transcriptomic markers of BPD risk.

## Methods

### Study cohort

The Discovery-Bronchopulmonary Dysplasia Program (D-BPD) cohort was described in previous publications^21,22^ and much of the methods overlap with the present work. In brief, in the parent study, 378 preterm infants < 1500 g of birth weight in Buenos Aires, Argentina, were recruited within 13 days of life and followed prospectively in the NICU until discharged or 44 weeks of corrected GA^21,22^. A diagnosis of BPD was made for infants who received at least 28 days of O_2_ (> 21%) supplementation therapy and need for O_2_ (≥ 30%) and/or positive pressure (1) at 36 weeks of PMA or at discharge (whichever comes first) if born < 32 weeks GA or (2) at 28–56 days postnatal age or at discharge (whichever comes first) if born ≥ 32 weeks GA, as defined in Jobe and Bancalari^23^. A total of 109 patients (15 BPD, 94 nonBPD) satisfying all study inclusion criteria provided a cord and/or peripheral blood sample for methylation arrays and/or RNA-Seq analysis (Table 1; Additional file 1: Table S1). The study methods were performed in accordance with the relevant guidelines and regulations and the design was approved by each of the local Institutional Review Boards (IRB) in Buenos Aires and at the NIEHS (08-E-N159). Parents provided written informed consent as described elsewhere^21,22^.

### Genomic DNA and total RNA extraction

Cord or peripheral blood samples were collected at birth and at day 14 and day 28 of life, placed in PAXgene reagent (Qiagen Inc., Valencia, CA) and then snap frozen at – 80 °C. The PAXgene Blood miRNA Kit (PreAnalytix/Qiagen) was used following the manufacturer’s procedure. As in Wang et al.^22^ blood specimens were incubated at room temperature for > 2 h to lyse RBCs and centrifuged (3500 g, 15 min) to acquire cell pellets. Pellets were washed and treated with proteinase K at 55 °C (800 rpm, 15 min), and isopropanol was added to the soluble fractions of the supernatants prepared from the QiaShredder spin columns. For RNA isolation, the isopropanol precipitants were added into the PAXgene RNA spin columns and processed for DNase treatment followed by RNA extraction procedures as indicated in the manufacturer’s brochure. As in Wang et al.^22^, for DNA isolation, the isopropanol precipitants prepared with the PAXgene miRNA Kit were loaded into the DNeasy Mini spin columns (DNeasy Blood & Tissue Kit, Qiagen) and followed the manufacturer’s procedure. DNAs and RNAs were quantified using Qubit (Thermo Fisher, Waltham, MA) and stored at – 80 °C until used.

### DNA methylation microarray analysis

Aliquots (100–500 ng) of genomic DNA from whole peripheral blood were bisulfite-converted using EZ-96 DNA Methylation MagPrep kits (Zymo Research, Irvine, CA) following the manufacturer’s instructions as described previously^22^. Bisulfite-converted DNAs were applied to Human MethylationEPIC BeadChip (Illumina, San Diego, CA), which covers over 850,000 CpG sites in the human genome, at the NIEHS Molecular Genomics Core Laboratory. The raw IDAT files from the EPIC methylation arrays were read into R with the minfi package^51^, and the data was preprocessed with background correction and dye-bias normalization using the preprocessNoob method^52^ as in^22^. The champ.runCombat function in ChAMP package^53^ was used for batch correction ("Sentrix_ID" and "Sentrix_Position"). DNA methylation data were filtered prior to normalization, using the following exclusion criteria: arrays with > 5% failed probes; CpG probes located on X and Y chromosomes; and any probes containing one or more single-nucleotide polymorphisms having a minor allele frequency ≥ 1% (in EUR population of the 1000 Genomes Project) occurring within 5 nucleotides relative to the CpG site. Probes reported to hybridize to one or more non-target sites in the genome were deleted^54^. There were 775,201 CpG probes remaining after exclusions. DNA methylation array data are deposited in Gene Expression Omnibus (GEO, accession number: GSE225313). To detect potential outlier samples in the methylation dataset we prepared principal component analysis (PCA) plots of methylation data from Day 14, Day 28 and combined (Additional file 2: Fig S6). No methylation samples were excluded.

### Methylation-based cord blood and peripheral blood cell type estimation

The percentages of seven blood cell types (CD4 + T, CD8 + T, NK cells, B cells, monocytes, granulocytes, and nucleated red blood cells) were estimated using reference DNA methylation profiles (https://github.com/immunomethylomics/FlowSorted.CordBloodCombined.450k) for cord blood and the IDOL deconvolution algorithm^25^. The 12 cell-type model of Salas et al.^28^ was used to differentiate naïve from memory B cells, CD4 T cells and CD8 T cells.

### Differentially methylated loci associated with BPD or NICU O_2_ exposure

Identification of differentially methylated probes was accomplished using a robust linear regression analysis of M values (log ratio of beta values) on disease status (BPD vs nonBPD-exposed to ≥ 1 day O_2_ in NICU) on methylation values from postnatal day 14 and day 28 with adjustment for infant sex, GA, BW, and seven estimated blood cell type proportions (CD4 + T, CD8 + T, NK, B, monocyte, granulocyte, NRBC). Differentially methylated loci associated with O_2_ exposure were identified on day 14 among nonBPD neonates (nonBPD-no O_2_ vs nonBPD- ≥ 14 days O_2_ in NICU) with adjustment for the 10 covariates indicated above. The Winsorize technique (https://www.rdocumentation.org/packages/DescTools/versions/0.99.44/topics/Winsorize) was used in an alternative EWAS analysis to test if outlier data points affected the resulting differentially methylated CpGs. We did 90% winsorization, that is the top 5% of the data is replaced by the value of the data at the 95th percentile and the value of the bottom 5% of the data is replaced by the value of the data at the 5th percentile. The re-analysis of all EWAS comparisons using the Winsorization method produced very similar results for differentially methylated CpGs at the FDR1% level (Additional file 1: Table S13). Differentially methylated CpGs were annotated to genes using the Illumina manifest and also by identifying the nearest transcription start site, which resulted in some CpGs being associated with more than one transcript.

### RNA-seq analysis

Aliquots of RNA (250 ng) were used to generate poly-adenylated RNA libraries with TruSeq Stranded Total RNA Ribo-Zero Human Gold kit (Illumina). Samples were indexed with NEXTfle-96 RNA-Seq Barcodes (Bioo-scientific, Austin, TX) and 75 bp single-end sequencing was performed on NovaSeq 6000 platform using S4 flow cell (Illumina) in the NIEHS Epigenomics and DNA Sequencing Core Laboratory. Raw sequencing reads (FASTQ files, 22–150 million reads per sample) were aligned to hg38 using STAR and gene counts were generated with featureCounts using the GENCODE version 39 annotation. Count matrix data were then imported to Partek Flow (Partek Inc., Chesterfield, MO) and PCA was used to visualize outlier samples (Additional File 2: Fig S7). One outlier was removed from the cord blood analysis. Quantification of transcript expression and differential expression analyses were performed using DESeq adjusting for nine covariates (GA, BW, sex, CD4 + T%, CD8 + T%, B%, monocyte%, granulocyte%, NRBC%). DEGs were determined between BPD and nonBPD on day 0 (cord blood) or between BPD and nonBPD exposed to any day(s) of O_2_ on days 14 and 28 (peripheral blood) with cutoff for significance at *p* < 0.05 and/or FDR-adjusted *p* < 0.1. DEGs between nonBPD exposed no NICU O_2_ and nonBPD exposed to ≥ 14 days NICU O_2_ with cutoff for significance at FDR-adjusted *p* < 0.01. Controlled hierarchical cluster analysis by disease status generated heatmaps showing a structure of DEG expression trends and partition of DEGs into different clusters using Partek Flow. RNA-Seq raw data are deposited in GEO (accession number: GSE220135).

### Pathway analyses

Enrichment of canonical pathways, functions, biological processes and diseases for the genes annotated to the differentially methylated loci or the DEGs were analyzed using Ingenuity Pathway Analysis (IPA, Qiagen), ToppGene Suite (https://toppgene.cchmc.org), Reactome Pathway Database (https://reactome.org) and/or David BioInformatics Resources (https://david.ncifcrf.gov) as described previously^22^. All *p* values of pathways and gene ontologies were corrected for multiple testing using the Benjamini–Hochberg method.

### Statistical analyses

Association between percentages of granulocyte and GA, lymphocytes and GA, or NLR and NICU O_2_ days were analyzed by linear regression analyses without covariates (GraphPad Prism 9, GraphPad Software, San Diego, CA). Student’s *t*-test, Fisher Exact, or Chi-square tests were used to determine differences between BPD and nonBPD for each cell type percentage, NLR, and population demographics (SigmaPlot 14.0, Systat Software, San Jose, CA).

### Ethics approval and consent to participate

All participating mothers provided written formed consent. The research was performed under the supervision and approval of the Institutional Review Board at recruiting centers in Argentina and National Institute of Environmental Health Sciences, National Institutes of Health (Protocol 08-E-N159).

## Supplementary Information

Supplementary Tables.

Supplementary Figures.

## Data Availability

The datasets analyzed during the current study are available in the GEO repository (https://www.ncbi.nlm.nih.gov/gds) with accession numbers GSE225313 (DNA methylation) and GSE220135 (RNA-Seq). The other datasets generated or analyzed during the current study are included in this article and its supplementary information files or available from the corresponding author on reasonable request.

## Abbreviations

AGER (or RAGE): Advanced glycosylation end-product receptor
AHRR: Aryl hydrocarbon receptor repressor
BPD: Bronchopulmonary dysplasia
BW: Birth weight
CTSH: Cathepsin H
D-BPD: The discovery-bronchopulmonary dysplasia program
DEG: Differentially expressed gene
EWAS: Epigenome-wide association study
FDR: False discovery rate
GA: Gestational age
GEO: Gene expression omnibus
GNLY: Granulysin
GO: Gene ontology
GWAS: Genome-wide association study
GZMA: Granzyme A
IDOL: Identifying optimal DNA methylation libraries
IFN: Interferon
IFITM: Interferon induced transmembrane protein
IL: Interleukin
IPA: Ingenuity pathway analysis
IRB: Institutional review board
LTF: Lactoferrin
MPO: Myeloperoxidase
NCOR2: Nuclear receptor corepressor 2
NICU: Neonatal intensive care unit
NIEHS: National Institute of Environmental Health Sciences
NIH: National Institutes of Health
NK: Natural killer cell
NLR: Neutrophil–lymphocyte ratio
NPIR: Negative regulator of interferon response
NRBC: Nucleated red blood cell
O_2_: Oxygen
PCA: Principal component analysis
PMA: Postmenstrual age
RAR: Retinoic acid receptor
RNA-Seq: RNA sequencing
ROP: Retinopathy of prematurity
ROS: Reactive oxygen species
SPOCK2: SPARC (osteonectin), Cwcv and Kazal like domains proteoglycan 2
TNFSF8: Tumor necrosis factor superfamily member 8
