# Supplementary material for: Prospective epigenome and transcriptome analyses of cord and peripheral blood from preterm infants at risk of bronchopulmonary dysplasia
Source: Sci Rep. 2023 Jul 28;13:12262. doi: 10.1038/s41598-023-39313-0 (PMC10382533; doi:10.1038/s41598-023-39313-0)
Supplement: Supplementary file 2 — Supplementary Figures. [file 41598_2023_39313_MOESM2_ESM.pptx]

## Slide 1
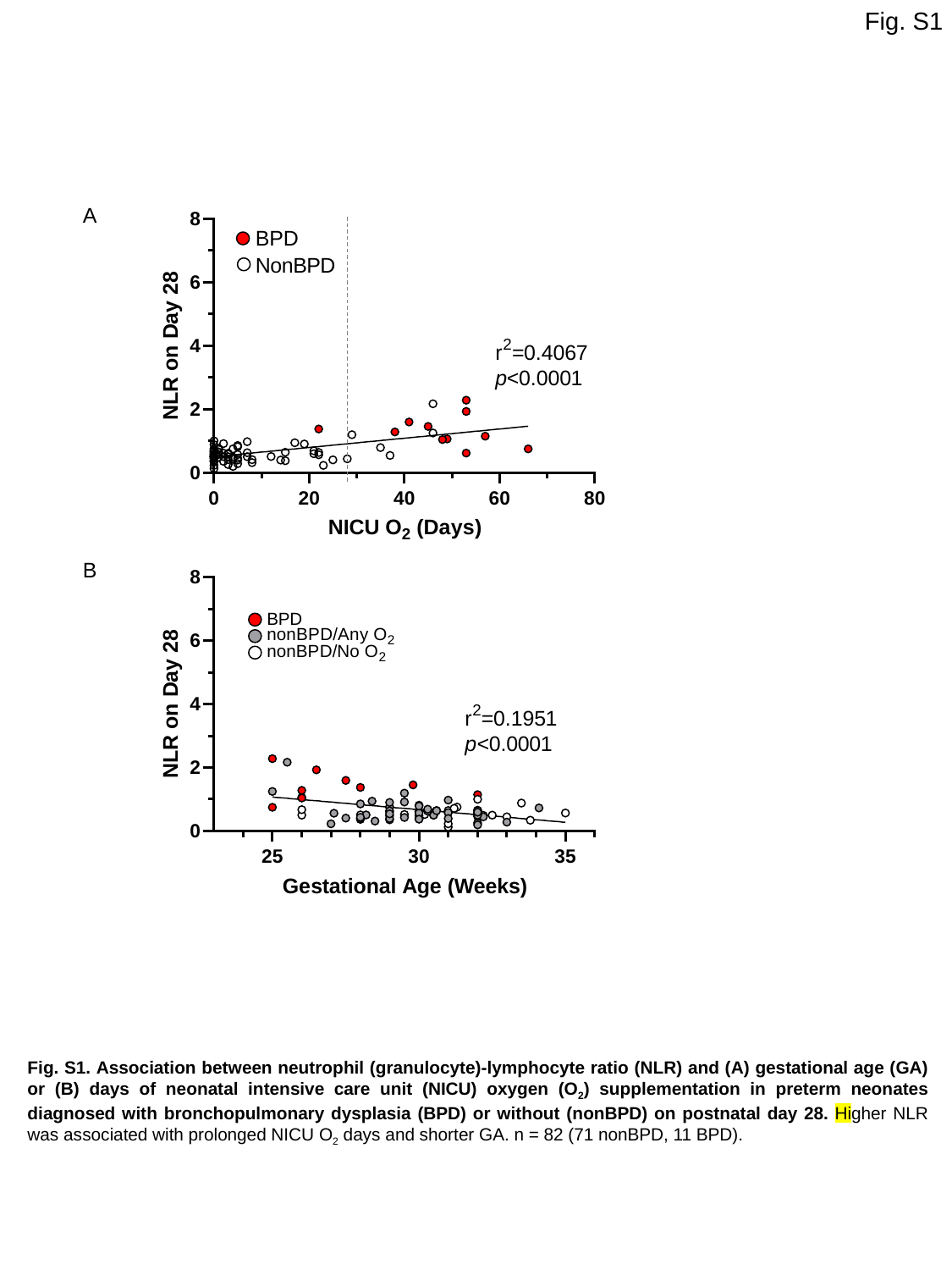

Fig. S1
A
B
Fig. S1. Association between neutrophil (granulocyte)-lymphocyte ratio (NLR) and (A) gestational age (GA) or (B) days of neonatal intensive care unit (NICU) oxygen (O2) supplementation in preterm neonates diagnosed with bronchopulmonary dysplasia (BPD) or without (nonBPD) on postnatal day 28. Higher NLR was associated with prolonged NICU O2 days and shorter GA. n = 82 (71 nonBPD, 11 BPD).

## Slide 2
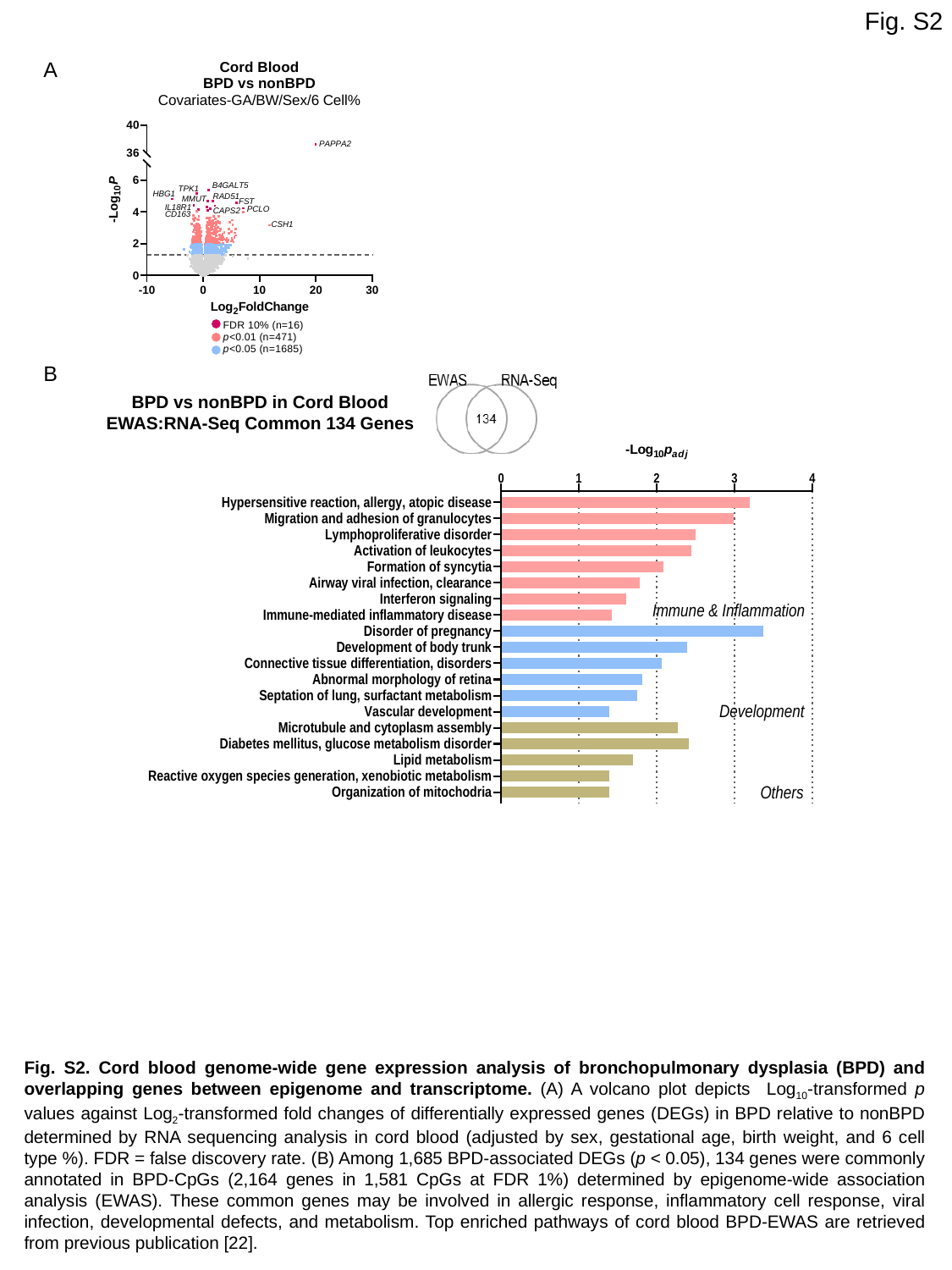

Fig. S2
A
B
BPD vs nonBPD in Cord Blood
EWAS:RNA-Seq Common 134 Genes
Immune & Inflammation
Development
Others
Fig. S2. Cord blood genome-wide gene expression analysis of bronchopulmonary dysplasia (BPD) and overlapping genes between epigenome and transcriptome. (A) A volcano plot depicts Log10-transformed p values against Log2-transformed fold changes of differentially expressed genes (DEGs) in BPD relative to nonBPD determined by RNA sequencing analysis in cord blood (adjusted by sex, gestational age, birth weight, and 6 cell type %). FDR = false discovery rate. (B) Among 1,685 BPD-associated DEGs (p < 0.05), 134 genes were commonly annotated in BPD-CpGs (2,164 genes in 1,581 CpGs at FDR 1%) determined by epigenome-wide association analysis (EWAS). These common genes may be involved in allergic response, inflammatory cell response, viral infection, developmental defects, and metabolism. Top enriched pathways of cord blood BPD-EWAS are retrieved from previous publication [22].

## Slide 3
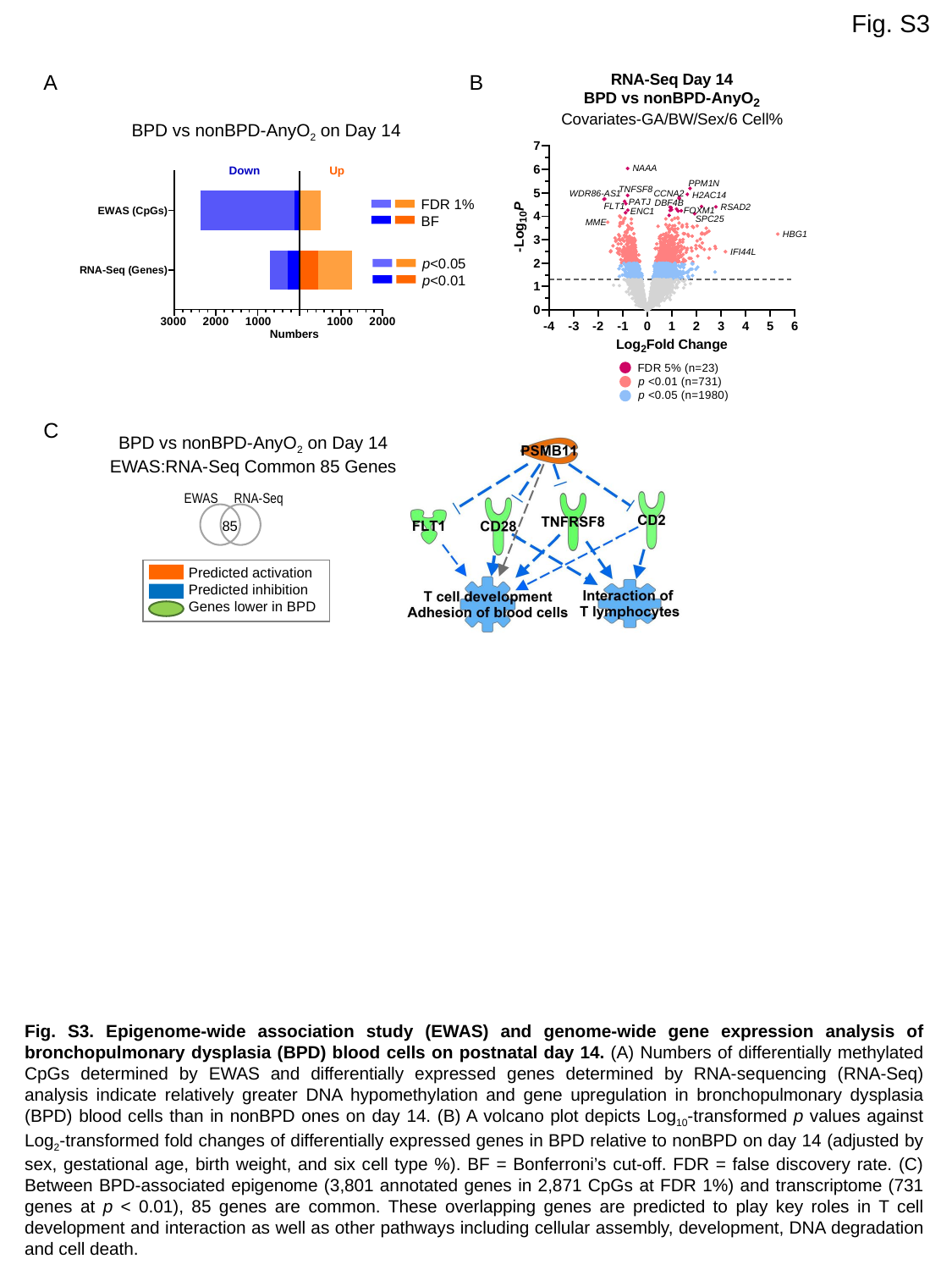

Fig. S3
A B
BPD vs nonBPD-AnyO2 on Day 14
FDR 1%
BF
p<0.05
p<0.01
C
BPD vs nonBPD-AnyO2 on Day 14
EWAS:RNA-Seq Common 85 Genes
EWAS RNA-Seq
85
Predicted activation
Predicted inhibition
Genes lower in BPD
Fig. S3. Epigenome-wide association study (EWAS) and genome-wide gene expression analysis of bronchopulmonary dysplasia (BPD) blood cells on postnatal day 14. (A) Numbers of differentially methylated CpGs determined by EWAS and differentially expressed genes determined by RNA-sequencing (RNA-Seq) analysis indicate relatively greater DNA hypomethylation and gene upregulation in bronchopulmonary dysplasia (BPD) blood cells than in nonBPD ones on day 14. (B) A volcano plot depicts Log10-transformed p values against Log2-transformed fold changes of differentially expressed genes in BPD relative to nonBPD on day 14 (adjusted by sex, gestational age, birth weight, and six cell type %). BF = Bonferroni’s cut-off. FDR = false discovery rate. (C) Between BPD-associated epigenome (3,801 annotated genes in 2,871 CpGs at FDR 1%) and transcriptome (731 genes at p < 0.01), 85 genes are common. These overlapping genes are predicted to play key roles in T cell development and interaction as well as other pathways including cellular assembly, development, DNA degradation and cell death.

## Slide 4
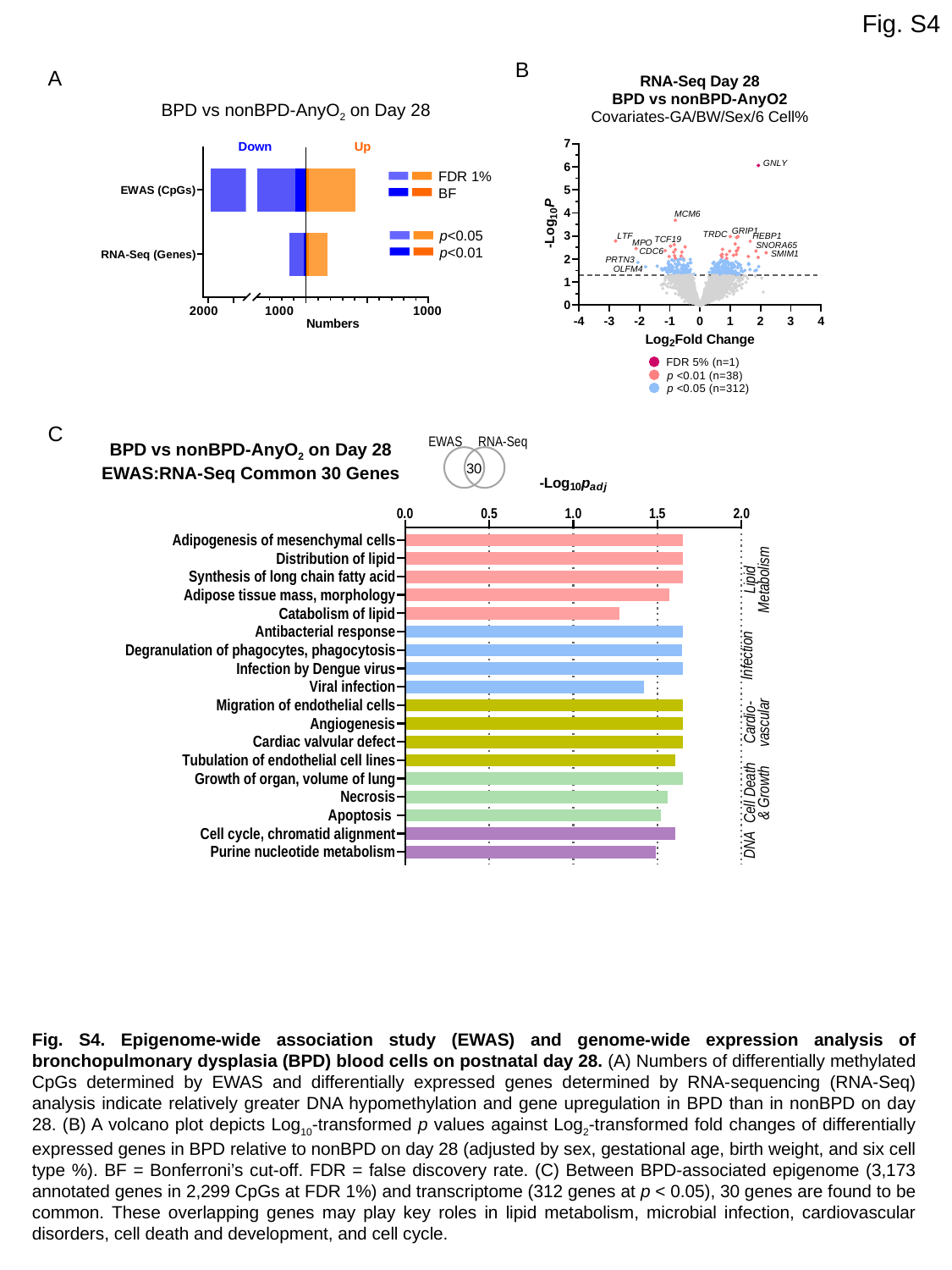

Fig. S4
B
A
C
BPD vs nonBPD-AnyO2 on Day 28
FDR 1%
BF
p<0.05
p<0.01
EWAS RNA-Seq
30
BPD vs nonBPD-AnyO2 on Day 28
EWAS:RNA-Seq Common 30 Genes
Lipid
Metabolism
Infection
Cardio-
vascular
Cell Death
& Growth
DNA
Fig. S4. Epigenome-wide association study (EWAS) and genome-wide expression analysis of bronchopulmonary dysplasia (BPD) blood cells on postnatal day 28. (A) Numbers of differentially methylated CpGs determined by EWAS and differentially expressed genes determined by RNA-sequencing (RNA-Seq) analysis indicate relatively greater DNA hypomethylation and gene upregulation in BPD than in nonBPD on day 28. (B) A volcano plot depicts Log10-transformed p values against Log2-transformed fold changes of differentially expressed genes in BPD relative to nonBPD on day 28 (adjusted by sex, gestational age, birth weight, and six cell type %). BF = Bonferroni’s cut-off. FDR = false discovery rate. (C) Between BPD-associated epigenome (3,173 annotated genes in 2,299 CpGs at FDR 1%) and transcriptome (312 genes at p < 0.05), 30 genes are found to be common. These overlapping genes may play key roles in lipid metabolism, microbial infection, cardiovascular disorders, cell death and development, and cell cycle.

## Slide 5
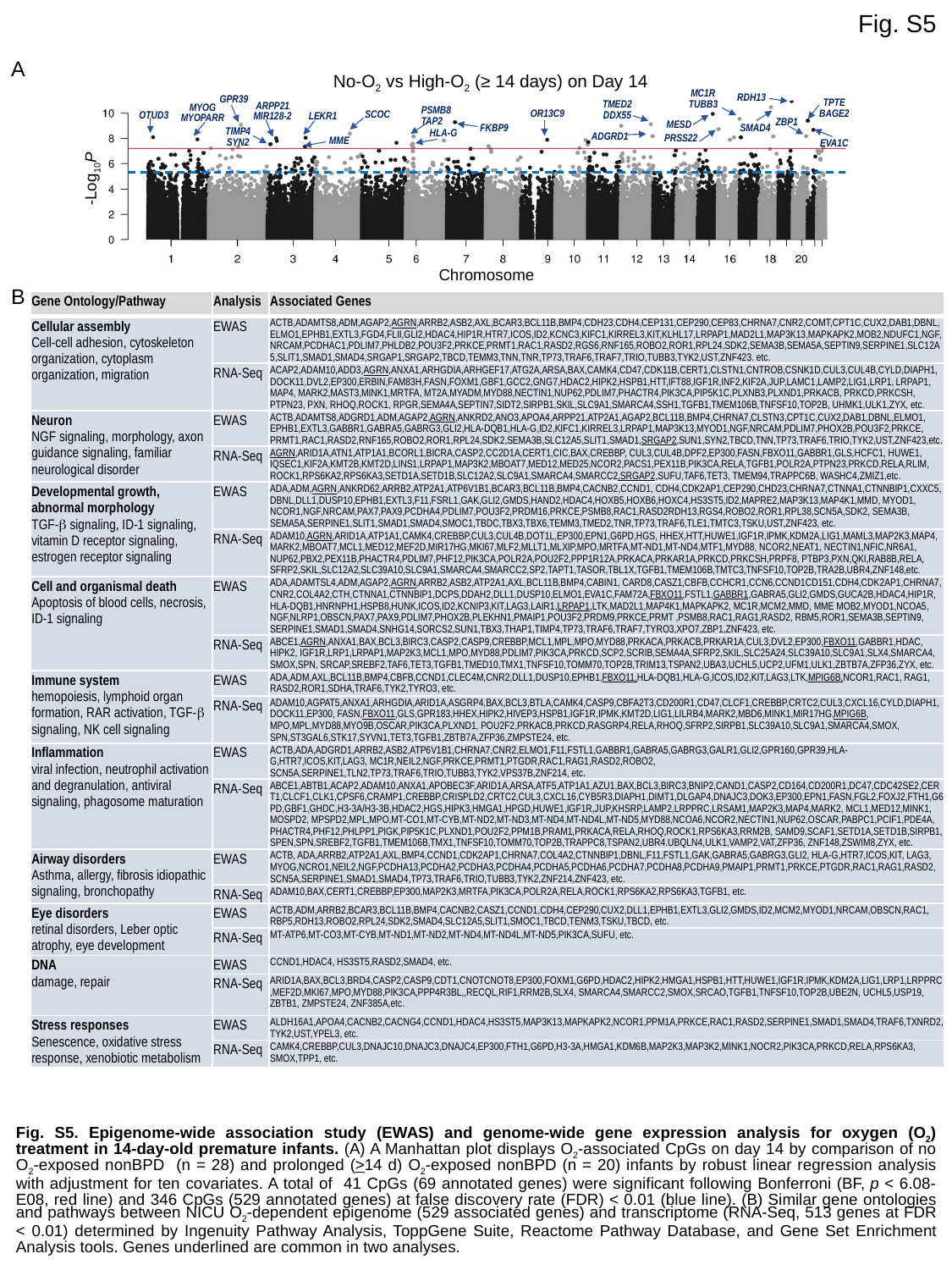

Fig. S5
-Log10P
Chromosome
MC1R
TUBB3
RDH13
GPR39
TPTE
BAGE2
TMED2
DDX55
ARPP21
MIR128-2
MYOG
MYOPARR
PSMB8
TAP2
OR13C9
SCOC
OTUD3
LEKR1
ZBP1
MESD
SMAD4
FKBP9
HLA-G
TIMP4
SYN2
ADGRD1
PRSS22
MME
EVA1C
No-O2 vs High-O2 (≥ 14 days) on Day 14
A
B
| Gene Ontology/Pathway | Analysis | Associated Genes |
| --- | --- | --- |
| Cellular assembly Cell-cell adhesion, cytoskeleton organization, cytoplasm organization, migration | EWAS | ACTB,ADAMTS8,ADM,AGAP2,AGRN,ARRB2,ASB2,AXL,BCAR3,BCL11B,BMP4,CDH23,CDH4,CEP131,CEP290,CEP83,CHRNA7,CNR2,COMT,CPT1C,CUX2,DAB1,DBNL,ELMO1,EPHB1,EXTL3,FGD4,FLII,GLI2,HDAC4,HIP1R,HTR7,ICOS,ID2,KCNC3,KIFC1,KIRREL3,KIT,KLHL17,LRPAP1,MAD2L1,MAP3K13,MAPKAPK2,MOB2,NDUFC1,NGF,NRCAM,PCDHAC1,PDLIM7,PHLDB2,POU3F2,PRKCE,PRMT1,RAC1,RASD2,RGS6,RNF165,ROBO2,ROR1,RPL24,SDK2,SEMA3B,SEMA5A,SEPTIN9,SERPINE1,SLC12A5,SLIT1,SMAD1,SMAD4,SRGAP1,SRGAP2,TBCD,TEMM3,TNN,TNR,TP73,TRAF6,TRAF7,TRIO,TUBB3,TYK2,UST,ZNF423. etc. |
| | RNA-Seq | ACAP2,ADAM10,ADD3,AGRN,ANXA1,ARHGDIA,ARHGEF17,ATG2A,ARSA,BAX,CAMK4,CD47,CDK11B,CERT1,CLSTN1,CNTROB,CSNK1D,CUL3,CUL4B,CYLD,DIAPH1,DOCK11,DVL2,EP300,ERBIN,FAM83H,FASN,FOXM1,GBF1,GCC2,GNG7,HDAC2,HIPK2,HSPB1,HTT,IFT88,IGF1R,INF2,KIF2A,JUP,LAMC1,LAMP2,LIG1,LRP1, LRPAP1, MAP4, MARK2,MAST3,MINK1,MRTFA, MT2A,MYADM,MYD88,NECTIN1,NUP62,PDLIM7,PHACTR4,PIK3CA,PIP5K1C,PLXNB3,PLXND1,PRKACB, PRKCD,PRKCSH, PTPN23, PXN, RHOQ,ROCK1, RPGR,SEMA4A,SEPTIN7,SIDT2,SIRPB1,SKIL,SLC9A1,SMARCA4,SSH1,TGFB1,TMEM106B,TNFSF10,TOP2B, UHMK1,ULK1,ZYX, etc. |
| Neuron NGF signaling, morphology, axon guidance signaling, familiar neurological disorder | EWAS | ACTB,ADAMTS8,ADGRD1,ADM,AGAP2,AGRN,ANKRD2,ANO3,APOA4,ARPP21,ATP2A1,AGAP2,BCL11B,BMP4,CHRNA7,CLSTN3,CPT1C,CUX2,DAB1,DBNL,ELMO1, EPHB1,EXTL3,GABBR1,GABRA5,GABRG3,GLI2,HLA-DQB1,HLA-G,ID2,KIFC1,KIRREL3,LRPAP1,MAP3K13,MYOD1,NGF,NRCAM,PDLIM7,PHOX2B,POU3F2,PRKCE, PRMT1,RAC1,RASD2,RNF165,ROBO2,ROR1,RPL24,SDK2,SEMA3B,SLC12A5,SLIT1,SMAD1,SRGAP2,SUN1,SYN2,TBCD,TNN,TP73,TRAF6,TRIO,TYK2,UST,ZNF423,etc. |
| | RNA-Seq | AGRN,ARID1A,ATN1,ATP1A1,BCORL1,BICRA,CASP2,CC2D1A,CERT1,CIC,BAX,CREBBP, CUL3,CUL4B,DPF2,EP300,FASN,FBXO11,GABBR1,GLS,HCFC1, HUWE1, IQSEC1,KIF2A,KMT2B,KMT2D,LINS1,LRPAP1,MAP3K2,MBOAT7,MED12,MED25,NCOR2,PACS1,PEX11B,PIK3CA,RELA,TGFB1,POLR2A,PTPN23,PRKCD,RELA,RLIM, ROCK1,RPS6KA2,RPS6KA3,SETD1A,SETD1B,SLC12A2,SLC9A1,SMARCA4,SMARCC2,SRGAP2,SUFU,TAF6,TET3, TMEM94,TRAPPC6B, WASHC4,ZMIZ1,etc. |
| Developmental growth, abnormal morphology TGF-b signaling, ID-1 signaling, vitamin D receptor signaling, estrogen receptor signaling | EWAS | ADA,ADM,AGRN,ANKRD62,ARRB2,ATP2A1,ATP6V1B1,BCAR3,BCL11B,BMP4,CACNB2,CCND1, CDH4,CDK2AP1,CEP290,CHD23,CHRNA7,CTNNA1,CTNNBIP1,CXXC5, DBNL,DLL1,DUSP10,EPHB1,EXTL3,F11,FSRL1,GAK,GLI2,GMDS,HAND2,HDAC4,HOXB5,HOXB6,HOXC4,HS3ST5,ID2,MAPRE2,MAP3K13,MAP4K1,MMD, MYOD1, NCOR1,NGF,NRCAM,PAX7,PAX9,PCDHA4,PDLIM7,POU3F2,PRDM16,PRKCE,PSMB8,RAC1,RASD2RDH13,RGS4,ROBO2,ROR1,RPL38,SCN5A,SDK2, SEMA3B, SEMA5A,SERPINE1,SLIT1,SMAD1,SMAD4,SMOC1,TBDC,TBX3,TBX6,TEMM3,TMED2,TNR,TP73,TRAF6,TLE1,TMTC3,TSKU,UST,ZNF423, etc. |
| | RNA-Seq | ADAM10,AGRN,ARID1A,ATP1A1,CAMK4,CREBBP,CUL3,CUL4B,DOT1L,EP300,EPN1,G6PD,HGS, HHEX,HTT,HUWE1,IGF1R,IPMK,KDM2A,LIG1,MAML3,MAP2K3,MAP4, MARK2,MBOAT7,MCL1,MED12,MEF2D,MIR17HG,MKI67,MLF2,MLLT1,MLXIP,MPO,MRTFA,MT-ND1,MT-ND4,MTF1,MYD88, NCOR2,NEAT1, NECTIN1,NFIC,NR6A1, NUP62,PBX2,PEX11B,PHACTR4,PDLIM7,PHF12,PIK3CA,POLR2A,POU2F2,PPP1R12A,PRKACA,PRKAR1A,PRKCD,PRKCSH,PRPF8, PTBP3,PXN,QKI,RAB8B,RELA, SFRP2,SKIL,SLC12A2,SLC39A10,SLC9A1,SMARCA4,SMARCC2,SP2,TAPT1,TASOR,TBL1X,TGFB1,TMEM106B,TMTC3,TNFSF10,TOP2B,TRA2B,UBR4,ZNF148,etc. |
| Cell and organismal death Apoptosis of blood cells, necrosis, ID-1 signaling | EWAS | ADA,ADAMTSL4,ADM,AGAP2,AGRN,ARRB2,ASB2,ATP2A1,AXL,BCL11B,BMP4,CABIN1, CARD8,CASZ1,CBFB,CCHCR1,CCN6,CCND1CD151,CDH4,CDK2AP1,CHRNA7, CNR2,COL4A2,CTH,CTNNA1,CTNNBIP1,DCPS,DDAH2,DLL1,DUSP10,ELMO1,EVA1C,FAM72A,FBXO11,FSTL1,GABBR1,GABRA5,GLI2,GMDS,GUCA2B,HDAC4,HIP1R, HLA-DQB1,HNRNPH1,HSPB8,HUNK,ICOS,ID2,KCNIP3,KIT,LAG3,LAIR1,LRPAP1,LTK,MAD2L1,MAP4K1,MAPKAPK2, MC1R,MCM2,MMD, MME MOB2,MYOD1,NCOA5, NGF,NLRP1,OBSCN,PAX7,PAX9,PDLIM7,PHOX2B,PLEKHN1,PMAIP1,POU3F2,PRDM9,PRKCE,PRMT ,PSMB8,RAC1,RAG1,RASD2, RBM5,ROR1,SEMA3B,SEPTIN9, SERPINE1,SMAD1,SMAD4,SNHG14,SORCS2,SUN1,TBX3,THAP1,TIMP4,TP73,TRAF6,TRAF7,TYRO3,XPO7,ZBP1,ZNF423, etc. |
| | RNA-Seq | ABCE1,AGRN,ANXA1,BAX,BCL3,BIRC3,CASP2,CASP9,CREBBP,MCL1,MPL,MPO,MYD88,PRKACA,PRKACB,PRKAR1A,CUL3,DVL2,EP300,FBXO11,GABBR1,HDAC, HIPK2, IGF1R,LRP1,LRPAP1,MAP2K3,MCL1,MPO,MYD88,PDLIM7,PIK3CA,PRKCD,SCP2,SCRIB,SEMA4A,SFRP2,SKIL,SLC25A24,SLC39A10,SLC9A1,SLX4,SMARCA4, SMOX,SPN, SRCAP,SREBF2,TAF6,TET3,TGFB1,TMED10,TMX1,TNFSF10,TOMM70,TOP2B,TRIM13,TSPAN2,UBA3,UCHL5,UCP2,UFM1,ULK1,ZBTB7A,ZFP36,ZYX, etc. |
| Immune system hemopoiesis, lymphoid organ formation, RAR activation, TGF-b signaling, NK cell signaling | EWAS | ADA,ADM,AXL,BCL11B,BMP4,CBFB,CCND1,CLEC4M,CNR2,DLL1,DUSP10,EPHB1,FBXO11,HLA-DQB1,HLA-G,ICOS,ID2,KIT,LAG3,LTK,MPIG6B,NCOR1,RAC1, RAG1, RASD2,ROR1,SDHA,TRAF6,TYK2,TYRO3, etc. |
| | RNA-Seq | ADAM10,AGPAT5,ANXA1,ARHGDIA,ARID1A,ASGRP4,BAX,BCL3,BTLA,CAMK4,CASP9,CBFA2T3,CD200R1,CD47,CLCF1,CREBBP,CRTC2,CUL3,CXCL16,CYLD,DIAPH1,DOCK11,EP300, FASN,FBXO11,GLS,GPR183,HHEX,HIPK2,HIVEP3,HSPB1,IGF1R,IPMK,KMT2D,LIG1,LILRB4,MARK2,MBD6,MINK1,MIR17HG,MPIG6B, MPO,MPL,MYD88,MYO9B,OSCAR,PIK3CA,PLXND1, POU2F2,PRKACB,PRKCD,RASGRP4,RELA,RHOQ,SFRP2,SIRPB1,SLC39A10,SLC9A1,SMARCA4,SMOX, SPN,ST3GAL6,STK17,SYVN1,TET3,TGFB1,ZBTB7A,ZFP36,ZMPSTE24, etc. |
| Inflammation viral infection, neutrophil activation and degranulation, antiviral signaling, phagosome maturation | EWAS | ACTB,ADA,ADGRD1,ARRB2,ASB2,ATP6V1B1,CHRNA7,CNR2,ELMO1,F11,FSTL1,GABBR1,GABRA5,GABRG3,GALR1,GLI2,GPR160,GPR39,HLA-G,HTR7,ICOS,KIT,LAG3, MC1R,NEIL2,NGF,PRKCE,PRMT1,PTGDR,RAC1,RAG1,RASD2,ROBO2, SCN5A,SERPINE1,TLN2,TP73,TRAF6,TRIO,TUBB3,TYK2,VPS37B,ZNF214, etc. |
| | RNA-Seq | ABCE1,ABTB1,ACAP2,ADAM10,ANXA1,APOBEC3F,ARID1A,ARSA,ATF5,ATP1A1,AZU1,BAX,BCL3,BIRC3,BNIP2,CAND1,CASP2,CD164,CD200R1,DC47,CDC42SE2,CERT1,CLCF1,CLK1,CPSF6,CRAMP1,CREBBP,CRISPLD2,CRTC2,CUL3,CXCL16,CYB5R3,DIAPH1,DIMT1,DLGAP4,DNAJC3,DOK3,EP300,EPN1,FASN,FGL2,FOXJ2,FTH1,G6PD,GBF1,GHDC,H3-3A/H3-3B,HDAC2,HGS,HIPK3,HMGA1,HPGD,HUWE1,IGF1R,JUP,KHSRP,LAMP2,LRPPRC,LRSAM1,MAP2K3,MAP4,MARK2, MCL1,MED12,MINK1, MOSPD2, MPSPD2,MPL,MPO,MT-CO1,MT-CYB,MT-ND2,MT-ND3,MT-ND4,MT-ND4L,MT-ND5,MYD88,NCOA6,NCOR2,NECTIN1,NUP62,OSCAR,PABPC1,PCIF1,PDE4A, PHACTR4,PHF12,PHLPP1,PIGK,PIP5K1C,PLXND1,POU2F2,PPM1B,PRAM1,PRKACA,RELA,RHOQ,ROCK1,RPS6KA3,RRM2B, SAMD9,SCAF1,SETD1A,SETD1B,SIRPB1, SPEN,SPN,SREBF2,TGFB1,TMEM106B,TMX1,TNFSF10,TOMM70,TOP2B,TRAPPC8,TSPAN2,UBR4.UBQLN4,ULK1,VAMP2,VAT,ZFP36, ZNF148,ZSWIM8,ZYX, etc. |
| Airway disorders Asthma, allergy, fibrosis idiopathic signaling, bronchopathy | EWAS | ACTB, ADA,ARRB2,ATP2A1,AXL,BMP4,CCND1,CDK2AP1,CHRNA7,COL4A2,CTNNBIP1,DBNL,F11,FSTL1,GAK,GABRA5,GABRG3,GLI2, HLA-G,HTR7,ICOS,KIT, LAG3, MYOG,NCRO1,NEIL2,NGF,PCDHA13,PCDHA2,PCDHA3,PCDHA4,PCDHA5,PCDHA6,PCDHA7,PCDHA8,PCDHA9,PMAIP1,PRMT1,PRKCE,PTGDR,RAC1,RAG1,RASD2, SCN5A,SERPINE1,SMAD1,SMAD4,TP73,TRAF6,TRIO,TUBB3,TYK2,ZNF214,ZNF423, etc. |
| | RNA-Seq | ADAM10,BAX,CERT1,CREBBP,EP300,MAP2K3,MRTFA,PIK3CA,POLR2A,RELA,ROCK1,RPS6KA2,RPS6KA3,TGFB1, etc. |
| Eye disorders retinal disorders, Leber optic atrophy, eye development | EWAS | ACTB,ADM,ARRB2,BCAR3,BCL11B,BMP4,CACNB2,CASZ1,CCND1,CDH4,CEP290,CUX2,DLL1,EPHB1,EXTL3,GLI2,GMDS,ID2,MCM2,MYOD1,NRCAM,OBSCN,RAC1, RBP5,RDH13,ROBO2,RPL24,SDK2,SMAD4,SLC12A5,SLIT1,SMOC1,TBCD,TENM3,TSKU,TBCD, etc. |
| | RNA-Seq | MT-ATP6,MT-CO3,MT-CYB,MT-ND1,MT-ND2,MT-ND4,MT-ND4L,MT-ND5,PIK3CA,SUFU, etc. |
| DNA damage, repair | EWAS | CCND1,HDAC4, HS3ST5,RASD2,SMAD4, etc. |
| | RNA-Seq | ARID1A,BAX,BCL3,BRD4,CASP2,CASP9,CDT1,CNOTCNOT8,EP300,FOXM1,G6PD,HDAC2,HIPK2,HMGA1,HSPB1,HTT,HUWE1,IGF1R,IPMK,KDM2A,LIG1,LRP1,LRPPRC,MEF2D,MKI67,MPO,MYD88,PIK3CA,PPP4R3BL,,RECQL,RIF1,RRM2B,SLX4, SMARCA4,SMARCC2,SMOX,SRCAO,TGFB1,TNFSF10,TOP2B,UBE2N, UCHL5,USP19, ZBTB1, ZMPSTE24, ZNF385A,etc. |
| Stress responses Senescence, oxidative stress response, xenobiotic metabolism | EWAS | ALDH16A1,APOA4,CACNB2,CACNG4,CCND1,HDAC4,HS3ST5,MAP3K13,MAPKAPK2,NCOR1,PPM1A,PRKCE,RAC1,RASD2,SERPINE1,SMAD1,SMAD4,TRAF6,TXNRD2,TYK2,UST,YPEL3, etc. |
| | RNA-Seq | CAMK4,CREBBP,CUL3,DNAJC10,DNAJC3,DNAJC4,EP300,FTH1,G6PD,H3-3A,HMGA1,KDM6B,MAP2K3,MAP3K2,MINK1,NOCR2,PIK3CA,PRKCD,RELA,RPS6KA3, SMOX,TPP1, etc. |
Fig. S5. Epigenome-wide association study (EWAS) and genome-wide gene expression analysis for oxygen (O2) treatment in 14-day-old premature infants. (A) A Manhattan plot displays O2-associated CpGs on day 14 by comparison of no O2-exposed nonBPD (n = 28) and prolonged (>14 d) O2-exposed nonBPD (n = 20) infants by robust linear regression analysis with adjustment for ten covariates. A total of 41 CpGs (69 annotated genes) were significant following Bonferroni (BF, p < 6.08-E08, red line) and 346 CpGs (529 annotated genes) at false discovery rate (FDR) < 0.01 (blue line). (B) Similar gene ontologies and pathways between NICU O2-dependent epigenome (529 associated genes) and transcriptome (RNA-Seq, 513 genes at FDR < 0.01) determined by Ingenuity Pathway Analysis, ToppGene Suite, Reactome Pathway Database, and Gene Set Enrichment Analysis tools. Genes underlined are common in two analyses.

## Slide 6
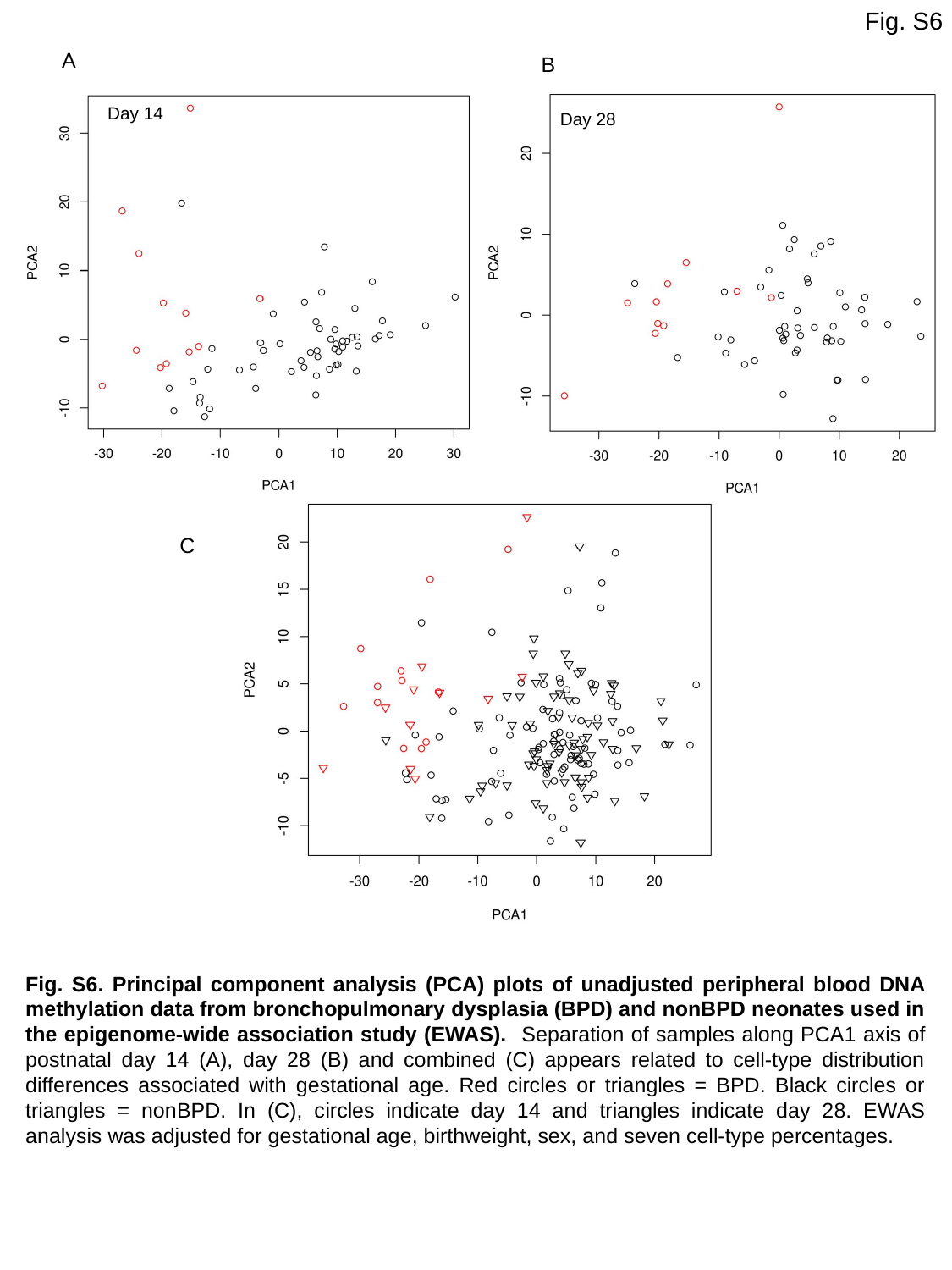

Fig. S6
A
B
Day 14
Day 28
C
Fig. S6. Principal component analysis (PCA) plots of unadjusted peripheral blood DNA methylation data from bronchopulmonary dysplasia (BPD) and nonBPD neonates used in the epigenome-wide association study (EWAS). Separation of samples along PCA1 axis of postnatal day 14 (A), day 28 (B) and combined (C) appears related to cell-type distribution differences associated with gestational age. Red circles or triangles = BPD. Black circles or triangles = nonBPD. In (C), circles indicate day 14 and triangles indicate day 28. EWAS analysis was adjusted for gestational age, birthweight, sex, and seven cell-type percentages.

## Slide 7
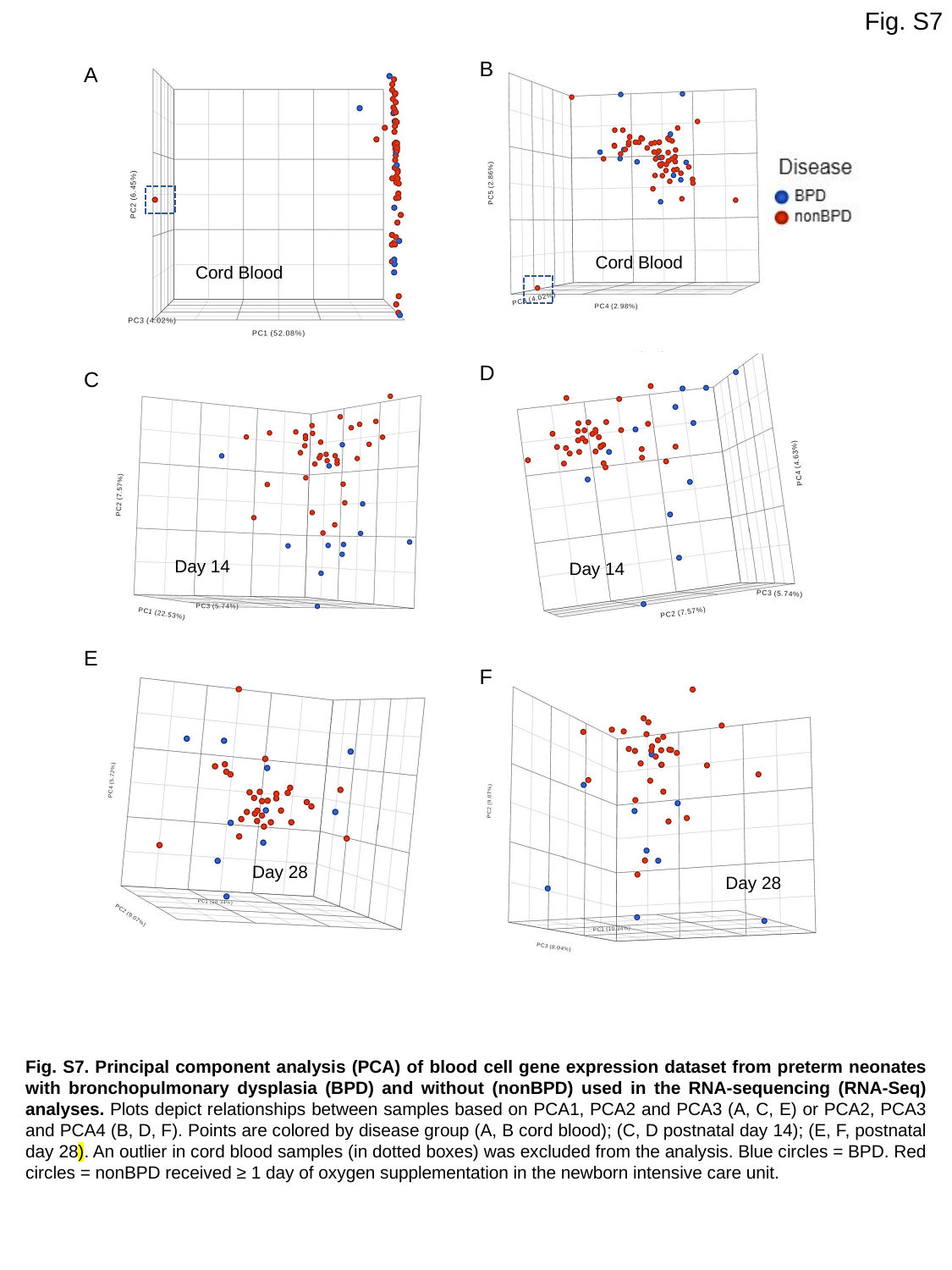

Fig. S7
B
D
F
A
C
E
Cord Blood
Cord Blood
Day 14
Day 14
Day 28
Day 28
Fig. S7. Principal component analysis (PCA) of blood cell gene expression dataset from preterm neonates with bronchopulmonary dysplasia (BPD) and without (nonBPD) used in the RNA-sequencing (RNA-Seq) analyses. Plots depict relationships between samples based on PCA1, PCA2 and PCA3 (A, C, E) or PCA2, PCA3 and PCA4 (B, D, F). Points are colored by disease group (A, B cord blood); (C, D postnatal day 14); (E, F, postnatal day 28). An outlier in cord blood samples (in dotted boxes) was excluded from the analysis. Blue circles = BPD. Red circles = nonBPD received ≥ 1 day of oxygen supplementation in the newborn intensive care unit.
